# Evaluation of computational genotyping of structural variation for clinical diagnoses

**DOI:** 10.1093/gigascience/giz110

**Published:** 2019-09-08

**Authors:** Varuna Chander, Richard A Gibbs, Fritz J Sedlazeck

**Affiliations:** Human Genome Sequencing Center, Baylor College of Medicine, 1 Baylor Plaza, Houston, TX 77030, USA

**Keywords:** structural variations, genotyping, clinical diagnosis, next-generation sequencing

## Abstract

**Background:**

Structural variation (SV) plays a pivotal role in genetic disease. The discovery of SVs based on short DNA sequence reads from next-generation DNA sequence methods is error-prone, with low sensitivity and high false discovery rates. These shortcomings can be partially overcome with extensive orthogonal validation methods or use of long reads, but the current cost precludes their application for routine clinical diagnostics. In contrast, SV genotyping of known sites of SV occurrence is relatively robust and therefore offers a cost-effective clinical diagnostic tool with potentially few false-positive and false-negative results, even when applied to short-read DNA sequence data.

**Results:**

We assess 5 state-of-the-art SV genotyping software methods, applied to short-read sequence data. The methods are characterized on the basis of their ability to genotype different SV types, spanning different size ranges. Furthermore, we analyze their ability to parse different VCF file subformats and assess their reliance on specific metadata. We compare the SV genotyping methods across a range of simulated and real data including SVs that were not found with Illumina data alone. We assess sensitivity and the ability to filter initial false discovery calls. We determined the impact of SV type and size on the performance for each SV genotyper. Overall, STIX performed the best on both simulated and GiaB based SV calls, demonstrating a good balance between sensitivity and specificty.

**Conclusion:**

Our results indicate that, although SV genotyping software methods have superior performance to SV callers, there are limitations that suggest the need for further innovation.

## Background

With the continuous advancement of sequencing technologies, our understanding of the importance of structural variation (SV) is increasing [1]. SV plays a critical role in evolution [2], genetic diseases (e.g., Mendelian or cancer) [2, 3], and the regulation of genes in different cells and tissues [4]. Furthermore, SVs constitute a substantial proportion of the genomic differences between cell types, individuals, populations, and species [1, 4–8]. SV is generally defined as 50 bp or longer genomic variation and is categorized into 5 types: insertions, deletions, duplications, inversions, and translocations [9]. SV is most often identified by leveraging combinations of paired-end, split read signals, and coverage information [8].

Methods for the *de novo* detection of SVs are still in their infancy, with some procedures reporting high (up to 89%) levels of false discovery [7, 8, 10–12] (i.e., SVs that are inferred due to artifacts but not truly present in the sample) and between 10% and 70% false-negative results [5, 7] (i.e., failure to find SVs that are present in the samples). Although deeper DNA sequence coverage is often used to improve *de novo* discovery of SVs, e.g., in cancer samples [13], this alone does not solve the sensitivity and accuracy shortcomings. The performance of these methods can be improved by the use of long DNA sequence reads; however, this is often not practical due to high sequencing costs [14–16]. Therefore, using short reads alone significantly hinders SV discovery for routine clinical diagnosis [17].

An additional challenge is the interpretation of the possible functional consequences of SVs. Despite the availability of existing methods to compare SVs (e.g., SURVIVOR [5]) and to study the potential impact of SVs on genes (VCFanno [18], SURVIVOR_ant [19]), there is still a paucity of methods to assess their allele frequency among human populations. These issues can hinder routine screening for SVs and limit their proper recognition and characterization for clinical diagnoses.

The identification of SVs that have been previously characterized in different samples is, in principle, easier than *de novo* detection. For known SVs it is possible to computationally detect their presence directly from short-read DNA sequence data from individual patient samples, guided by the expected position of split reads and discordant paired reads that can confirm breakpoints. This less demanding approach reduces false discovery rates and therefore renders the methods more suitable for clinical applications. In addition, the false-negative rate can be reduced because it is easier to genotype a variant than to identify a new SV. Focusing on known SVs has further the advantage, compared with *de novo* discovery of SVs, that SV databases will have likely recorded the event, together with its possible association with disease (e.g., dbVar [20]).

Here, we review the current state of SV genotyping methods and investigate their potential for application in clinical diagnoses. In particular, we address whether these SV calling software programs (“SV genotypers”) can re-identify SVs that short-read *de novo* SV callers failed to identify (over Genome in a Bottle [GIAB] [21, 22] call sets) and how they perform on initially falsely inferred SVs. We describe which SV genotypers most efficiently identify which types of SVs and the effect of SV size.

## Analyses

### Existing methods

We assessed 5 SV genotypers: DELLY [23], Genome STRiP [24], STIX [25], SV2 [26], and SVTyper [27]. They share a common feature in that they require a bam file of the mapped reads and a VCF file that will be genotyped for SVs as inputs. Table 1 lists their dependencies and their ability to genotype certain types of SVs.

**Table 1:** Overview of the SV genotypers and their ability to assess different SV types

Genotyper	Approach	SV type					Inputs	Dependencies
		Deletion	Insertion	Inversion	Duplication	Translocation/BND		
**Delly**	RD, PR, SR	✓		*	*	*	BAM, VCF, Ref	Bcftools [30]
**Svtyper**	SR, PR	✓		✓	✓	*	BAM, VCF, Ref	
**SV2**	RD, PR, SR	✓			✓		BAM, SNV VCF, VCF, Ref, PED file	
**STIX**	PR, SR	✓		✓			BAM compressed, PED file, VCF, Ref	Excord, Giggle [31]
**Genome StRiP**	RD, PR, SR	✓			✓		BAM, VCF, Ref	GATK [32]

✓ : works on a standardized VCF file.

* : marks dependencies on specialized tags in the VCF files. PR: paired-end reads; RD: read depth; SR: split reads.

Overall, they can be divided into groups that support only two SV types (e.g., Genome STRiP) up to methods that support all SV types (SVTyper and DELLY) but require specific meta-information. In the following, we give a brief description of each method that we assessed. Further insights can be obtained from their respective publications or manuals.

DELLY [23] is originally an SV caller that includes a genotype mode to redefine multi-sample VCFs. It operates on split and paired-end reads to genotype deletions, duplications, inversions, and translocations. However, for all types except the deletions, DELLY requires a sequence resolved call in its own format to be able to estimate the genotype.

Genome STRiP [24] genotypes only deletions and duplications. The unique aspect of Genome STRiP is that it was designed to genotype multiple samples simultaneously. It requires the GATK pipeline and prepackaged reference metadata bundles.

STIX [25], which is the most recently developed method included here, uses a reverse approach to the previous two examples. First, STIX extracts the discordant read pairs and split reads and generates a searchable index per sample. This index can then be queried if it supports a specific variant call. Noteworthy, STIX in the current form only provides information on how many reads support a variant rather than the genotype itself. This is done with a flag describing whether the reads are supported by a particular variant and the number of reads supporting it.

SVTyper [27] uses a Bayesian likelihood model that is based on discordant paired-end reads and split reads. It was designed to genotype deletions, duplications, inversions, and translocations. For the latter, however, SVTyper requires specific ID tags provided by Lumpy [28] to complete genotyping.

SV2 [26] uses a support vector machine learning to genotype deletions and duplications based on discordant paired-end, split read, and coverage. Furthermore, it was the only SV genotyper assessed here that leverages single-nucleotide polymorphism (SNP) calls for its predictions.

### Evaluation of SV computational genotypers based on simulated data

To first assess the performance of genotyping methods for SVs, we simulated data sets with 100 bp Illumina-like paired-end reads. Each data set includes 20 homozygous SVs simulated for a certain SV type (duplications, indels, inversions, and translocation) and a certain size range (100, 250, and 500 bp and 1, 2, 5, 10, and 50 kb). For each of the data sets, we called SVs using SURVIVOR [5] based on a union set of DELLY, Manta [29], and Lumpy [28] calls to include true-positive as well as false-positive SV calls (see Methods).

We discovered only 17 false-positive calls after the initial SV discovery. This low number of false-positive results is in contrast to reports from other studies. However, here we are using simulated data that do not take into account the complexities involved in regions of SVs and other sequencing biases. Interestingly, while this simulated data set represents an ideal case, we still missed ∼17.25% of the simulated SVs.

Supplementary Table 1 shows the results for the SV discovery set over the 32 simulated data sets based on 640 simulated SVs on chr21 and chr22.

**Figure 1:**
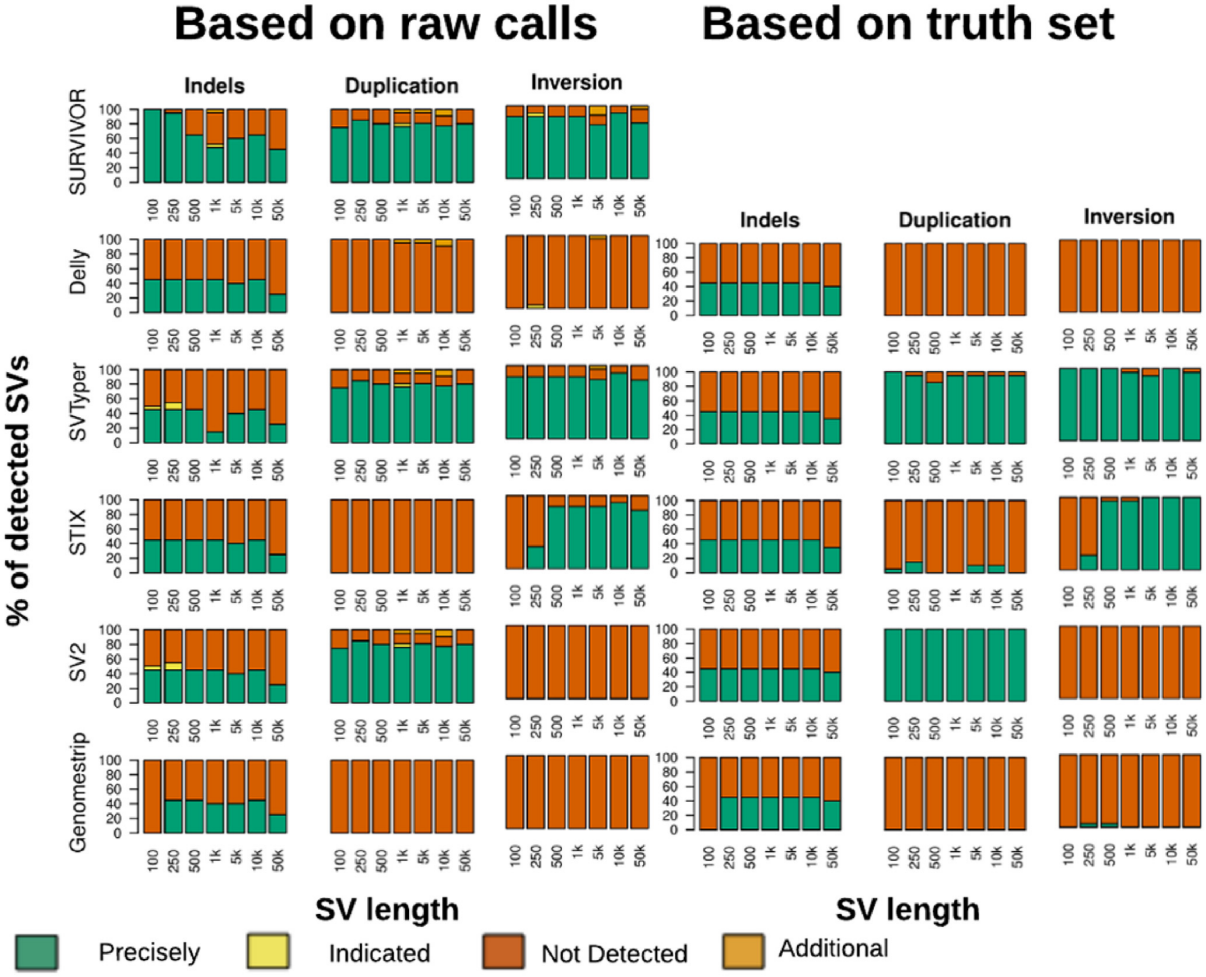
Evaluation of Illumina-like reads to assess the SV genotyper ability to re-identify insertions, deletions, duplications, and inversions over different size ranges (x-axis). The colors indicate the SVs being detected/genotyped by the respective SV genotypers. They were classified as either precisely, indicated, not detected, or falsely identified (see Methods). For the SVs genotyped on the basis of SV calls (left) we used SURVIVOR, which is a union set of Delly, Lumpy, and Manta, to generate the VCF file as an input for the SV genotypers. Noteworthy, Delly and SVtyper can genotype more SVs, given the custom information from their respective callers, Delly and SVTyper, respectively. When the truth SV set is provided as a start point (right panel) we see marginal improvements across the SV genotyping methods while maintaining the overall trend.

The generated VCF files were taken as input for the 5 SV genotyper callers: DELLY, Genome STRiP, SV2, STIX, and SVTyper. Fig. 1 provides an overview with respect to the ability to discover SVs in the first place (SURVIVOR). We did not visualize translocations/BND because none of the genotypers were able to identify them based upon our standard conform VCF file. Supplementary Table 1 shows the results for all SV genotypers, applied to the 32 simulated data sets.

Interestingly, we observed that certain methods require a specialized VCF file with information provided specific to one SV caller. For example, while SVTyper is able to genotype deletions, inversions, and duplications, it will work on BND (translocation) events only if the ID pairs provided by Lumpy are included in the VCF file. Additionally, DELLY, which is capable of inferring deletion, inversion, duplication, and translocation types of SVs, is only able to genotype deletions if using a standardized VCF without the extra information.

The overall performance of each method was evaluated on the basis of the input VCF generated by SURVIVOR. Thus, if all of the short-read–based SV callers were not able to resolve the insertions of 5 kb, then they would be assessed as a “wrong/missed” SV.

First, we assessed the ability of the SV genotypers to correctly genotype SVs. SVTyper (64.70%) had the highest rate of correctly genotyping SVs to be present, followed by SV2 (41.57%). Importantly, SV2 was able to genotype deletions and duplications, while SVTyper assessed deletions, duplications, and inversions. Genome STRiP had the lowest (14.40%) success rate of all methods because it can only genotype deletions and duplications. This result was expected because Genome STRiP was designed primarily for population-based genotyping. SVTyper improved marginally (86.26%) when BND events, which represented translocations, were ignored, followed by the next best method SV2 (83.15%) when focused on deletions and duplications. Furthermore, we also benchmarked the SV genotyping methods on their performance, given the truth set (Supplementary Table 2). The different methods show performance differences in the runtime ranging from 0.3 seconds (STIX) to 33.8 minutes (Genome STRiP) (Supplementary Table 3).

Next, we assessed the ability of the SV genotypers to reduce the rate of false-positive results, i.e., initially wrongly inferred SVs. This represents the scenario of accidentally genotyping an SV that is not represented in the sample due to sequencing or mapping biases. Over the 32 call sets, SURVIVOR had only 17 false-positive calls for the simulated data. Genome STRiP performed best in filtering out all falsely detected SVs but had the lowest ability to genotype SV variations. STIX performed better because it could filter out 13 (76.4%) of the false-positive SV calls. In contrast, STIX also achieved a higher (71.76%) performance for correctly identifying SVs. Although SVTyper had the most accurately genotyped SVs, it filtered out fewer false-positive results (70.59%) obtained during the discovery phase.

In summary, we observed that none of the methods were clearly superior for correctly genotyping and correctly filtering/non-reporting SV variation. Strikingly, none of the programs were able to genotype insertions or translocations in the simulated data sets. Nevertheless, STIX and SV2 showed strong performance, with a good balance of sensitivity and ability to correctly discard false-positive results.

#### Evaluation of SV computational genotypers based on GIAB Ashkenazi Son

We further assessed genotyping of SV calls based on the long-read DNA sequence data from an “Ashkenazi Son” (HG002) reference sample. Specifically, we tested the currently released call set (v0.5.0) from GIAB, generated using sequence resolved calls from multiple technologies such as Illumina, Pacific Biosciences (PacBio), and Bionano Genomics (BioNano) and multiple SV callers and *de novo* assemblies based on these technologies, alone or in combination [21]. It is important to note that 8,195 of these SV calls could not be initially discovered with any Illumina assembly or caller but originated from PacBio-based calls or BioNano-based calling.

We next used this call set to genotype the SVs based on a 300x Illumina bam file for HG002 and compare the obtained SV genotype predictions with the genotypes reported by GIAB. The first observation was that most of the SV genotypers were unable to process the VCF file provided by GIAB. We used SURVIVOR to reduce the information included in the GIAB VCF file. Next, we filtered out the reported insertions and complex events from this call set because most SV genotypers failed computationally to complete assessing these entries. Unfortunately, we were not able to run Genome STRiP successfully because it repeatedly failed, even when applied to just a subset of these calls.

**Figure 2:**
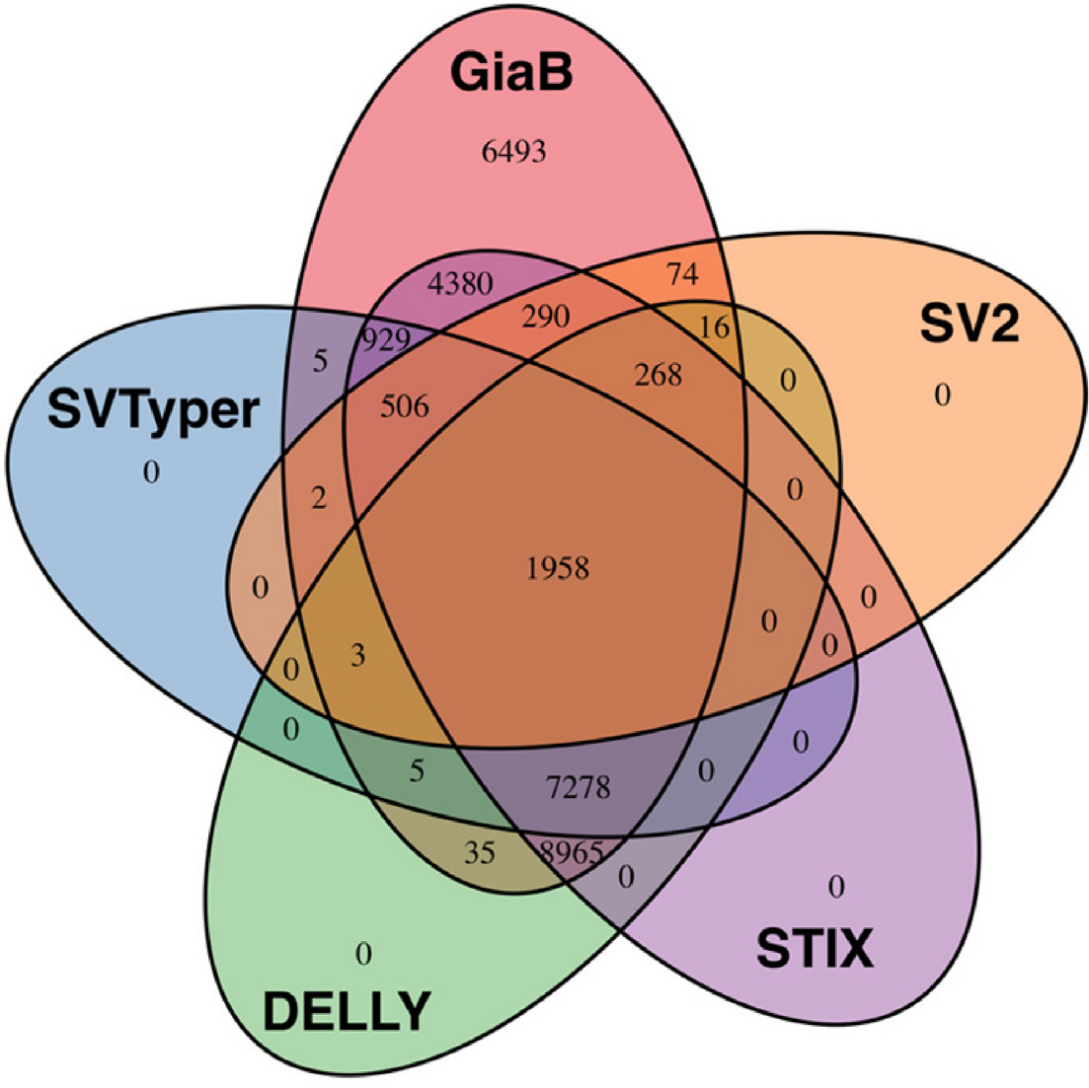
Evaluation based on GIAB call set v0.5.0 deletions only.

Fig. 2 displays the detectable deletions based on the GIAB call set (v0.5.0) per SV genotyper. STIX performed the best among all methods, identifying 24,574 (78.74%) of the provided deletions. It is important to note that STIX does not currently report genotypes. Thus, we relied only on the information if STIX found any single read that support this SV rather than genotype information. DELLY performed second best, identifying 18,528 (59.37%) deletions, followed by SVTyper (34.24%) and SV2 (9.99%). Only 6.27% of the deletion calls from the GIAB call set were genotyped by all SV genotype methods. Although this is a very low percentage, it is encouraging that up to 78.74% of the deletions could be successfully identified out of 31,207 deletions (≥20 bp) in total. Noteworthy, 4,921 deletions out of this set were never observed by any Illumina-based caller or assembly. This highlights the potential benefit of using SV genotypers.

Next, we assessed the size range in which SV genotypers were able to recognize SVs. The deletions from GIAB call set 0.5.0 ranged from 20 bp up to 997 kb, with a median size of 36 bp. All of the SV genotypers were able to identify deletions down to a size of 20 bp. Interestingly we observed different median sizes of genotyped deletions, which represents the ability of specific methods to resolve small vs large events. DELLY (31 bp) had the lowest median SV size, followed by SVTyper (32 bp), STIX (35 bp), and SV2 (116 bp). Furthermore, DELLY (816 kb) also genotyped the longest SVs, followed by STIX (694 kb), SV2 (656 kb), and SVTyper (656 kb). See Supplementary Table 4 for details.

When assessing the genotype concordance (see Supplementary Table 5), DELLY performed the best, with an agreement rate of 87.08% given that it identified the variant in the first place. SV2 achieved a 78.59% rate of genotype agreement; however, it had one of the lowest recall rates (9.99%). SVTyper showed a 67.79% genotype concordance. We did not evaluate STIX in this parameter because it does not report a genotype estimation in its current version.

In summary, STIX and DELLY performed the best in re-identifying the deletions reported by GIAB for HG0002. Furthermore, DELLY (87.08%) also had the highest agreement about the genotypes with the GIAB call set.

## Discussion

In this article, we assessed the current state of SV genotyping methods. These methods are valuable for identifying the genotype of SVs in new samples, at sites of already known validated and functionally annotated SVs. The methods are important for diagnostic applications because they offer better accuracy and reproducibility for the clinic than *de novo* detection methods.

An important observation was that as a practical matter, many SV genotypers are limited to applications linked to their *de novo* SV caller counterpart. For example, DELLY successfully genotyped all SV types subsequent to its use as a discovery method, but only when supplied with the DELLY-specific VCF file. Similarly, SVTyper relies on specific IDs associated with translocations (in this case BND) events provided by Lumpy.

We provided the first assessments of sensitivity and false discovery rate for SV genotypers that include not only Illumina-detectable SVs but those that could only be initially discovered via long-read technologies such as PacBio or Oxford Nanopore [14, 16]. These technologies often enable the detection of more complex SVs and those within regions that are difficult to resolve by Illumina alone—but are neither scalable or accurate enough to support routine *de novo* SV identification in a clinical setting [17].

This study also identified both general and method-specific limitations of SV genotyping methods. First, we observed that none of the methods tested was able to assess novel insertions that also represent repeat expansions, which is a subclass of SVs recognized as important in cancer and other diseases. Second, most of the methods are hindered by strict VCF formatting requirements, ignoring the current standard conventions, relying on individual flags that are difficult to emulate.

Among the SV genotypers, STIX performed best when applied to simulated and GIAB-based SV calls, demonstrating a good balance of high sensitivity vs reduced false discovery rate with the added ability to use standard VCF files. Nevertheless, the lack of genotype estimations for STIX remains a limitation. In aggregate, our results indicate that SV genotypers perform better than SV callers. Our approach can be integrated into existing analysis pipelines for routine scanning of known pathogenic SVs, representing an efficient and quick way to identify patients with SVs in the clinic.

## Potential Implications

SV genotyping represents an opportunity to infer SVs in clinical diagnostic settings where low false discovery and false-negative rates are critical. However, genotyping SV methods seem to require additional development to improve their ability to operate on different size events and on all types of SVs (including insertions). Here we present an overview of the state of the art and highlight the need for specific methodological improvements.

## Methods

### Simulated data sets

We simulated 20 SVs per data set each for a certain type (indel, inversions, duplication, and translocation) and a certain size (100, 250, and 500 bp and 1, 2, 5, 10, and 50 kb) for chr21 and chr22 using SURVIVOR simSV. These simulations included a 1% SNP rate. After the simulation of the sample genomes we simulated reads using Mason [33] with the parameters "Illumina -ll 500 -n 100 –N 39 773 784 -sq -mp -rn 2" to generate 100-bp paired-end Illumina-like reads. The reads were mapped with BWA MEM [34] using the –M option to mark duplicated reads to the entire genome (GRCh38–2.1.0). Subsequently, we ran Manta (v1.2.1), DELLY (v0.7.8), and Lumpy (v0.2.13) to call SVs over the simulated data sets. For each data set we generated a union call set based on all 3 callers using SURVIVOR merge (v1.0.3) allowing 1 kb distance and allowing only the same SV type to be merged. To assess the performance of the SV genotypers across the SV truth set, we used the output of SURVIVOR that was used for the evaluation. Subsequently, we converted that output to a VCF file using SURVIVOR bed2vcf. We incorporated CPOS and CIEND with both 0,0 to enable running SVTyper.

This union set, as well as the SV genotyper output, was evaluated with SURVIVOR eval for the following categories: Precise: calling an SV within 10 bp and inferring the correct type; Indicated: allowing a maximum of 1 kb between the simulated and the called breakpoints and ignoring the predicted type of SVs; Missing: a simulated SV but not re-identified; Additional: an SV that was called but not simulated. The results were summarized using a custom R script operating on the output of SURVIVOR available on request.

The runtime of each method was measured across all simulated data sets using Linux time, and the average CPU time was reported.

### SV genotyping: simulated data

For genotyping the simulated data set, we used the union call VCF based on the SURVIVOR output as described above. We used DELLY (v0.7.8) specifying the output (-o), the vcf to be genotyped (-v), and the reference file (-g) as fasta and the bam file. We ran DELLY with the VCF file from SURVIVOR over the SV discovery caller. The obtained output from DELLY was converted using bcftools view (v1.7 [using htslib 1.7]) [30] to obtain a VCF file and was filtered to ignore genotyped calls with 0/0. SVTyper (v0.1.4) was used on the VCF generated from SURVIVOR based on the discovery phase. We filtered the obtained VCF for genotypes that could not have been accessed by SVTyper. SV2 (version 1.4.3) was run on the SURVIVOR-generated VCF file for SV genotyping but also required a single-nucleotide variant (SNV) file. We generated this SNV file using Freebayes (v1.1.0-46-g8d2b3a0-dirty) [35] with the default parameters. The resulting SNV file from Freebayes was compressed and indexed by bgzip and tabix –p vcf [36], respectively. SV2 reports its result in 3 folders (sv2_preprocessed, sv2_features, and sv2_genotypes) from which we used the result reported in sv2_genotypes to benchmark the method. Genome STRiP (v2.00.1774) was used following the suggested parameters and the VCF file generated by SURVIVOR. STIX (early version available via GitHub on 6 April 2018) was used to index the bam file using giggle (v0.6.3) [31], excord (v0.2.2), and samtools (v1.7) [30] following the suggested pipeline. Next, we ran STIX with “-s 500” on the VCF files from SURVIVOR and ignoring output VCF entries with "STIX_ZERO = 1," which filters out entries where STIX does not find any evidence for the SV.

### SV genotyping: GIAB

We obtained the GIAB SV call set (v0.5.0) [37], the GIAB gold standard SNV calls [38], and the corresponding bam file [39] from the GIAB FTP. The SV call set needed to be filtered and reduced for just 1 sample (HG002) using cat and SURVIVOR and was subsequently filtered for deletions only. We ran all SV genotyping methods as described above. Subsequently, we filtered the results for genotypes: 0/1 and 1/1 with the exception of STIX. STIX was filtered on the basis of whether it reports reads to support the SVs or not. This was necessary because STIX does not currently report genotypes. After filtering we merged all data sets together including the original VCF provided using SURVIVOR with a maximum distance of 10 bp and requiring the same SV types. We analyzed these merged calls on the basis of whether the original call set reported a genotype to be heterozygous or homozygous alternative. The Venn diagram was generated on the basis of the support vector reported by SURVIVOR and the R package Venn.diagram. The length of the SVs that were able to be genotyped were extracted using awk filtering for existing calls.

## Availability of supporting data and materials

Data sets and scripts were deposited in the *GigaScience* Database, GigaDB [40]. We obtained the GIAB SV call set (v0.5.0) [37], the GIAB gold standard SNV calls [38], and the corresponding bam file [39] from the GIAB FTP.

## Additional files

GIGA-D-19-00035_Tables.xlsx.

Supplementary Table 1. Results for SV discovery set over 32 simulated data sets using SV *de novo* calls based on SURVIVR (Delly, Manta and Lumpy)

Supplementary Table 2. Results for SV discovery set over 32 simulated data sets using the ground truth.

Supplementary Table 3. Runtime of the different SV genotypers

Supplementary Table 4. Size distributions of genotyped SV based on GIAB v0.5

Supplementary Table 5. Agreement of genotypes per SV genotyper for GIAB v0.5 predictions

giz110_GIGA-D-19-00035_Original_Submission

giz110_GIGA-D-19-00035_Revision_1

giz110_GIGA-D-19-00035_Revision_2

giz110_Response_to_Reviewer_Comments_Original_Submission

giz110_Response_to_Reviewer_Comments_Revision_1

giz110_Reviewer_1_Report_Original_Submission David Quigley -- 2/28/2019 Reviewed

giz110_Reviewer_2_Report_Original_Submission Ryan Layer -- 3/19/2019 Reviewed

giz110_Reviewer_2_Report_Revision_1 Ryan Layer -- 7/22/2019 Reviewed

giz110_Supplemental_Tables

## Abbreviations

bp: base pairs; BWA: Burrows-Wheeler Aligner; CPU: central processing unit; GATK: Genome Analysis Toolkit; Genome STRiP: Genome STRucture In Populations; GIAB: Genome in a Bottle; kb: kilobase pairs; PacBio: Pacific Biosciences; SNP: single-nucleotide polymorphism; SNV: single-nucleotide variant; STIX: STructural variant IndeX; SV: structural variation;.

## Competing interests

F.J.S. has participated in PacBio and Oxford Nanopore sponsored meetings over the past few years and has received travel reimbursement and honoraria for presenting at these events.

## Funding

This research was supported by a National Institutes of Health award (UM1 HG008898).

## Authors’ contributions

V.C. and F.J.S. performed the analysis. All authors wrote the manuscript. F.J.S. and R.A.G. directed the project.
