# Supplementary material for: Evaluation of computational genotyping of structural variation for clinical diagnoses
Source: Gigascience. 2019 Sep 8;8(9):giz110. doi: 10.1093/gigascience/giz110 (PMC6732172; doi:10.1093/gigascience/giz110)
Supplement: giz110_GIGA-D-19-00035_Original_Submission [file giz110_giga-d-19-00035_original_submission.pdf]

GigaScience

Evaluation of computational genotyping of Structural Variations for clinical diagnoses

--Manuscript Draft--

**Manuscript Number:**	GIGA-D-19-00035
**Full Title:**	Evaluation of computational genotyping of Structural Variations for clinical diagnoses
**Article Type:**	Research
**Funding Information:**	National Human Genome Research Institute (UM1 HG008898) — Dr. Richard A. Gibbs
**Abstract:**	Background<br><br>In recent years, many Structural Variations (SVs) have been identified as having a pivotal role in causing genetic disease. Nevertheless, the discovery of SVs based on short DNA sequence reads from next-generation DNA sequence methods is still error-prone, suffering from low sensitivity and high false discovery. These shortcomings can be partially overcome with the use of long reads, but the current expense precludes their application for routine clinical diagnostics. SV genotyping, on the other hand, offers cost-effective application as diagnostic tool in the clinic, with potentially no false positives and low occurrence of false negatives.<br><br>Results<br><br>We assess five state- of-the- art SV genotyping software methods that test short read sequence data. These methods are characterized based on their ability to genotype certain SV types and size ranges. Furthermore, we analyze their applicability to parse different VCF file sub-formats, or to rely on specific meta information that is not always at hand. We compare the SV genotyping methods across a range of simulated and real data including SVs that were not found with Illumina data alone. We assess their sensitivity and ability to filter out initial false discovery calls to assess their reliability.<br><br>Conclusion<br><br>Our results indicate that, although SV genotypers have superior performance to SV callers, there are performance limitations that suggest the need for further innovation.
**Corresponding Author:**	Fritz Sedlazeck<br><br>UNITED STATES
**Corresponding Author Secondary Information:**	
**Corresponding Author's Institution:**	
**Corresponding Author's Secondary Institution:**	
**First Author:**	Varuna Chander
**First Author Secondary Information:**	
**Order of Authors:**	Varuna Chander<br>Richard A. Gibbs<br>Fritz J. Sedlazeck
**Order of Authors Secondary Information:**	
**Additional Information:**	
**Question**	**Response**
Are you submitting this manuscript to a	No

Powered by Editorial Manager® and ProduXion Manager® from Aries Systems Corporation

special series or article collection?	
**Experimental design and statistics**<br><br>Full details of the experimental design and statistical methods used should be given in the Methods section, as detailed in our Minimum Standards Reporting Checklist. Information essential to interpreting the data presented should be made available in the figure legends.<br><br>Have you included all the information requested in your manuscript?	Yes
**Resources**<br><br>A description of all resources used, including antibodies, cell lines, animals and software tools, with enough information to allow them to be uniquely identified, should be included in the Methods section. Authors are strongly encouraged to cite Research Resource Identifiers (RRIDs) for antibodies, model organisms and tools, where possible.<br><br>Have you included the information requested as detailed in our Minimum Standards Reporting Checklist?	Yes
**Availability of data and materials**<br><br>All datasets and code on which the conclusions of the paper rely must be either included in your submission or deposited in publicly available repositories (where available and ethically appropriate), referencing such data using a unique identifier in the references and in the "Availability of Data and Materials" section of your manuscript.<br><br>Have you have met the above requirement as detailed in our Minimum Standards Reporting Checklist?	Yes

Powered by Editorial Manager® and ProduXion Manager® from Aries Systems Corporation

Powered by Editorial Manager® and ProduXion Manager® from Aries Systems Corporation

Manuscript Click here to download Manuscript Manuscript.docx

Evaluation of computational genotyping of Structural Variations for clinical diagnoses.

Varuna Chander[1], Richard A. Gibbs[1], Fritz J. Sedlazeck[1]*

1: Human Genome Sequencing Center, Baylor College of Medicine, One Baylor Plaza, Houston TX 77030

VC: Varuna.Chander@bcm.edu

RG: agibbs@bcm.edu

FJS: fritz.sedlazeck@bcm.edu

* Correspondence: fritz.sedlazeck@bcm.edu

Abstract

Background:

In recent years, many Structural Variations (SVs) have been identified as having a pivotal role in causing genetic disease. Nevertheless, the discovery of SVs based on short DNA sequence reads from next-generation DNA sequence methods is still error-prone, suffering from low sensitivity and high false discovery. These shortcomings can be partially overcome with the use of long reads, but the current expense precludes their application for routine clinical diagnostics. SV genotyping, on the other hand, offers cost-effective application as diagnostic tool in the clinic, with potentially no false positives and low occurrence of false negatives.

Results:

We assess five state- of-the- art SV genotyping software methods that test short read sequence data. These methods are characterized based on their ability to genotype certain SV types and size ranges. Furthermore, we analyze their applicability to parse different VCF file sub-formats, or to rely on specific meta information that is not always at hand. We compare the SV genotyping methods across a range of simulated and real data including SVs that were not found with Illumina data alone. We assess their sensitivity and ability to filter out initial false discovery calls to assess their reliability.

Conclusion:

Our results indicate that, although SV genotypers have superior performance to SV callers, there are performance limitations that suggest the need for further innovation.

Keywords

Structural Variations, Genotyping, clinical diagnosis, Next Generation Sequencing

Background

With the continuous advancement of sequencing technologies, our understanding of the importance of Structural Variations (SVs) is increasing[1]. Structural Variations play critical roles in evolution[2], genetic diseases (e.g. mendelian or Cancer) [2, 3] and the regulation of cells/tissues[4]. Furthermore, SVs compromise a substantial proportion of genomic differences between cell types, individuals, populations and species [1, 4-8]. Structural Variations are generally identified as being 50bp or longer genomic variations and categorized into five types:

Insertions, Deletions, Duplications, Inversions and Translocations [9]. They are most often identified by leveraging paired-end, split read signals and coverage information[8].

Methods for the detection of SVs are still in their infancy, with some procedures reporting high (up to 89%) levels of false discovery[7, 8, 10-12] (i.e. SVs that are inferred due to artifacts, but not truly present in the sample) and between 10% to 70% false negatives[5, 7] (i.e. missing present SVs in the samples). Although the performance of these methods can improve by the use of long reads, this is often not practical due to high sequencing costs [13-15]. Therefore, using short reads alone significantly hinders SV discovery for routine clinical diagnosis [16].

An additional challenge is the interpretation of the possible functional consequences of SVs. Despite the availability of existing methods to compare SVs (e.g. SURVIVOR [5]) and to study the potential impact of SVs on genes (VCFanno [17], SURVIVOR_ant [18]), there is still a paucity of methods to assess their allele frequency among the human populations. These issues lead to problems that hinder routine screening for SVs in patient data and limit their proper recognition and characterization for clinical diagnoses.

The identification of SVs that have been previously identified in other, different samples is in principle, easier than *de novo* detection. For known SVs it is possible to computationally detect SVs directly from short read DNA sequence data in individual per patient samples, guided by the expected position of split reads and discordant paired reads that can confirm breakpoints. This is less demanding than calling *de novo* SVs, since we focus only on specific genomic

regions. This approach reduces the false discovery rates and therefore, reduces the potential misdiagnosis of patients. In addition, the false negative rate can be reduced as it is easier to genotype a variant than to identify a new SV. Genotyping known SVs has further the advantage that previous studies are likely to have annotated the event, and its possible association with certain diseases. Such events are recorded in SV databases (e.g. dbVar [19]).

In this paper, we review the current state of SVs genotyping methods and investigate their potential applicability for clinical diagnosis. In particular, we address whether these SV genotypers can re-identify SVs that short read callers were initially blind to (over GiaB [20] call sets) and how they perform for initially falsely inferred SVs. We precisely map out which genotypers operate on which types of SVs and their ability to genotype SVs based on sizes.

Analyses

Existing methods

Here we assess SVs genotyping methods: DELLY [21], Genome STRiP [22], STIX[23], SV2 [24] and SVTyper [25]. They share a common feature in which they require a bam file of the mapped reads and a VCF file that will be genotyped for SVs as inputs. **Table 1** lists their dependencies and their ability to genotype certain types of SVs.

Overall, they can be divided into groups that support only two SV types (e.g. Genome STRiP) up to methods that support all SV types (SVTyper and DELLY), but require specific meta-

information to do so. In the following, we give a brief description of each method that we assessed. Further insights can be obtained from their respective publications or manuals.

Genotyper	Approach	SV Type					Inputs	Dependencies
		Del	Ins	Inv	Dup	Tra		
**Delly**	RD, PR, SR	✓		✓	✓	✓	BAM, VCF, Ref	Bcftools [26]
**Svtyper**	SR, PR	✓		✓	✓	✓	BAM, VCF, Ref	
**SV2**	RD, PR, SR	✓			✓		BAM, SNV VCF, VCF, Ref, PED file	
**STIX**	PR,SR	✓		✓			BAM compressed, PED file, VCF, Ref	Excord, Giggle [27]
**Genome StRiP**	RD, PR, SR	✓			✓		BAM, VCF, Ref	GATK[28]

Table 1: Overview of the SV genotypers assessed here and their ability to assess different SV types. RD: read depth, SR: split reads, PR: paired end reads

DELLY[21] is originally an SV caller that includes a genotype mode to redefine multi-sample VCFs. It operates on split and paired-end reads to genotype deletions, duplications, inversions

and translations. However, for all types except the deletions, DELLY requires a sequence resolved call in its own format to be able to estimate the genotype.

Genome STRiP[22] genotypes only deletions and duplications. The unique aspect of Genome STRiP is that it was designed to genotype multiple samples simultaneously. It requires the GATK pipeline and prepackaged reference metadata bundles.

STIX[23], which is the most recently developed method included here, is designed the other way around. First, it extracts the discordant read pairs and split reads and generates a searchable index per sample. This index can then be queried if it supports a particular variant. Noteworthy, STIX in the current form only provides information on how many reads support a variant rather than the genotype itself. This is done with a flag describing whether the reads are supported by a particular variant and the number of reads supporting it.

SVTyper[25] uses a Bayesian likelihood model that is based on discordant paired-end reads and split reads. It was designed to genotype deletions, duplications, inversions and translocations. However, for the latter, it requires specific ID tags provided by Lumpy[29] to genotype them.

SV2[24] uses a support vector machine learning to genotype deletions and duplications based on discordant paired-end, split read and coverage. Furthermore, it was the only SV genotyper assessed here that leverages SNP calls for its prediction.

Evaluation of SVs computational genotypers based on simulated data

To first assess the performance of genotyping methods for SVs, we simulated data sets with 100bp Illumina like paired-end reads. Each data set includes 20 SVs simulated for a certain SVs type (duplications, indels, inversions and translocation) and a certain size range (100bp, 250bp, 500bp, 1kbp, 2kbp, 5kbp, 10kbp, 50kbp). For each of the data sets, we called SVs using SURVIVOR[5] based on a union set of DELLY, Manta[30], Lumpy[29] calls to include true positive as well as false positive SVs calls (see methods). Given the nature of the simulated data, we only observed 17 false discoveries while we were missing 17.25% of the simulated SVs. **Supplementary Table 1** shows the results for the discovery set over the 32 simulated data sets based on 640 simulated SVs on chr21 and chr22.

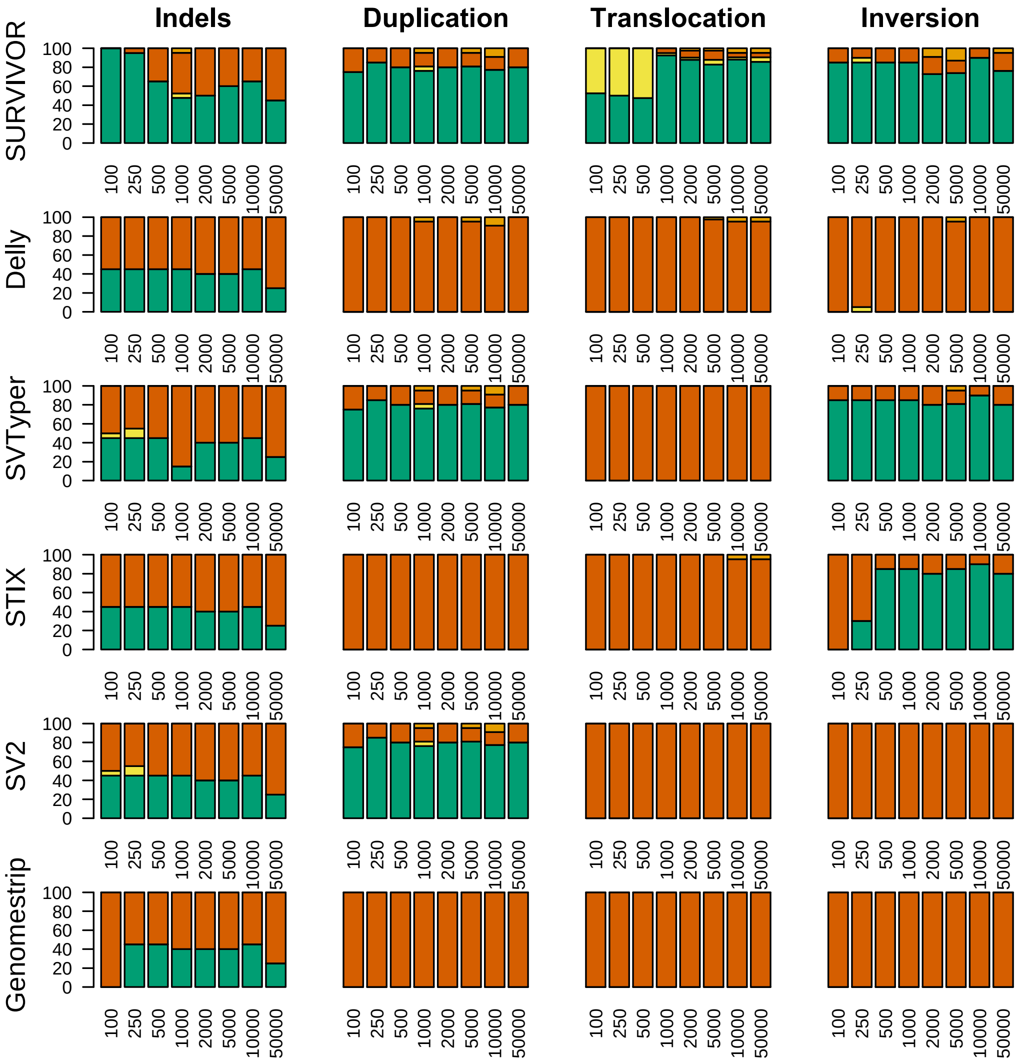

Figure 1: Evaluation of Illumina like reads to assess the SV genotyper ability to re-identify certain types and size ranges. SURVIVOR is a union set of Delly, Lumpy and Manta to generate the VCF file as an input for the SV genotypers. The evaluation is based on the ability to discover SVs in the first place (SURVIVOR). Noteworthy, Delly and SVtyper can genotype more SVs given their costume information provided from their caller which are Delly and SVTyper, respectively.

The generated VCF files were taken as input for five SV genotyper callers: DELLY, Genome STRiP, SV2, STIX and SVTyper. **Figure 1** provides an overview with respect to the ability to discover SVs in the first place (SURVIVOR). **Supplementary Table 1** shows the result for all genotypers over the 32 simulated data sets. Interestingly, we observed that not all methods

accept a standardized VCF file and certain SV types require unique information. For example, while SVTyper is able to genotype deletions, inversion and duplications, it will just work on BND (translocation) events if the ID pairs provided by Lumpy are included in the VCF file. Also DELLY, which is also capable to infer deletions, inversion, duplications and translocations types of SVs is only able to genotype deletions given a standardized VCF without the extra information.

The overall performance of each method was evaluated based on the input VCF generated by SURVIVOR. Thus, if all of the short read based SV callers were not able to resolve the insertions of 5kbp, then it would count as wrong/missed identified SV. In addition, we assessed the ability for the SV genotyper to filter out falsely called SVs. SVTyper (64.70%) had the highest rate of correctly genotyping SVs to be present, followed by SV2 (41.57%). Importantly, SV2 was able to genotype deletions and duplications, while SVTyper assessed deletions, duplications and inversions. Genome STRiP had the lowest (13.40%) success rate of all methods because it can only genotype deletions and duplications. One possible reason to keep in mind for this is that Genome STRiP was designed for population-based genotyping. SVTyper improved marginally (86.26%) when BND events, which represented translocations, were ignored, followed by the second best method SV2 (83.15%) when focused on deletions and duplications.

Next, we assessed the ability of the SV genotypers to reduce the false positives, i.e. initially wrongly inferred SVs. This will represent the scenario of accidentally genotyping a SV that is not represented in the sample due to sequencing or mapping biases. Over the 32 call sets, SURVIVOR had only 17 false positive calls for the simulated data. Genome STRiP performed best

with filtering out all falsely detected SVs, but suffers from the lowest ability to genotype SV variations. STIX performed better as it can filter out 13 (76.47%) of the false positive SV calls. In contrast, STIX also achieved a higher (71.76%) performance for correctly identifying SVs. Although SVTyper had the highest accurately genotyped SVs it performed poorly by filtering out 81.82% of the false positives obtained during the discovery phase.

In summary, we observed that none of the methods is clearly superior for correctly genotyping and correctly filtering/non-reporting variations. Especially non of the program were able to genotype insertions in the simulated data sets. Nevertheless, STIX and SV2 showed a good performance with a good balance of sensitivity and being able to correctly discard false positives.

Evaluation of SVs computational genotypers based on GiaB Ashkenazy Son

We further assessed the ability to genotype SVs calls based on the long read DNA sequence data from the Ashkenazy Son (HG002). We are using the current released call set (v0.5.0) from GiaB, which was generated using sequence resolved calls from multiple technologies such as Illumina, PacBio, BioNano etc. and multiple SV callers and *de novo* assemblies based on these technologies or a combination of them [20]. Here we are giving this call set the benefit of doubt. It is important to note that 8,195 of these SV calls could not be initially discovered with any Illumina assembly or caller but originated from PacBio based calls or BioNano based calling. We are using this call set to genotype the SVs based on a 300x Illumina bam file for HG002 and compare the obtained SV genotype predictions to the genotypes reported by GiaB. The first

observation was that most of the SV genotypers were unable to process the VCF file provided by GiaB. We used SURVIVOR to reduce the information included in the GiaB VCF file. Next, we filtered out the reported INS and complex events from this call set as most SV genotypers crashed while assessing these entries. Unfortunately, we were not able to run GenomeSTRiP successfully as it kept crashing even on a subset of these calls.

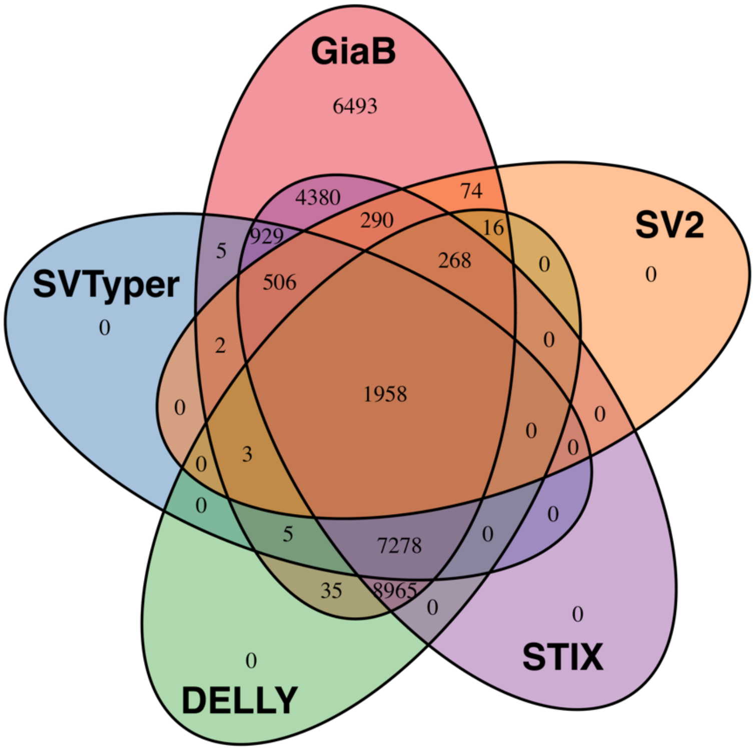

Figure 2: Evaluation based on GiaB call set v0.5.0 deletions only.

Figure 2 displays the detectable deletions based on the GiaB call set (v0.5.0) per SV genotyper. STIX performed the best among all methods identifying 24,574 (78.74%) of the provided

deletions. It is important to note that STIX does not currently report genotypes. Thus, we relied only on the information if STIX found reads that support this event rather than genotype information. DELLY performed as the second best identifying 18,528 (59.37%) deletions followed by SVTyper (34.24%) and SV2 (9.99%). Only 6.27% of the deletion calls from GiaB call set were genotyped by all SV genotype methods. Although this is a very low percent, it is positive that up to 78.74% of the deletions could be successfully identified out of 62,676 deletions (20bp+) in total. Noteworthy, 4921 deletions out of this set were never observed by any Illumina based caller or assembly. This highlights the potential benefit of using SV genotypers.

Next, we assessed the size regimes that SVs genotypers were able to recognize SVs (**Supplementary Table 2**). The deletions from GiaB call set 0.5.0 ranged from 20bp up to 997kbp with a median size of 36bp. All of the SV genotypers were able to identify deletions down to a size of 20bp. Interestingly we observed different median sizes of genotyped deletions, which represents the ability of specific methods to genotype small or large events. DELLY (31bp) had the lowest median SV size followed by SVTyper (32bp), STIX (35bp) and SV2 (116bp). Furthermore, DELLY (816kbp) genotyped also the longest SVs followed by STIX (694kbp), SV2 (656bkbp) and SVTyper (656bkbp).

When assessing the genotype concordance (**Supplementary Table 3**), DELLY performed the best with an agreement rate of 87.08% given that it identified the variant in the first place. SV2 achieved a 78.59% of genotype agreement, however it had one of the lowest recall rates

(9.99%). SVTyper showed a 67.79% genotypes concordance. We did not evaluate STIX in this perspective since it does not report a genotype estimation in its current version.

In summary, STIX and DELLY performed the best in re-identifying the deletions reported by GiaB for HG0002. Furthermore, DELLY (87.08%) had also the highest agreement over the genotypes with the GiaB call set.

Discussion

In this paper, we assessed the current state of SV genotyping methods. These methods are essential not just in assigning genotypes to discovery methods, but represents the ability to genotype already known, validated and functional annotated SVs. This latter aspect is important for clinical applications as it represents an early opportunity to reliably assess relevant SVs in the clinic.

Unfortunately, we discovered that many SV genotypers are only designed for their de novo SV caller counterpart in mind to report genotype estimations. This is especially obvious for DELLY, which can genotype all types subsequent to its discovery method, but only works on deletions based on a standardized VCF file. The same behavior, although reduced, can be observed by SVTyper that relies on specific IDs associated to translocations (in this case BND) events provided by Lumpy.

We were able to establish the first assessments of sensitivity and false discovery rate for SV genotypers not only focusing on Illumina detectable SVs, but further for SVs that were impossible to be discovered by Illumina alone. The latter becomes important as the field is continuously identifying SVs that based on long read technologies such as PacBio or Oxford Nanopore [13, 15]. These technologies often enable the detection of more complex SVs and also the detection of variations in regions that are hard to assess by Illumina alone. Thus, we rely on SV genotyper to assess if a particular SVs is present either in experiments with large sample sizes to evaluate the allele frequency or in clinical screenings to ease the diagnosis of patients [16].

Unfortunately, our study also highlights multiple general issues of SV genotyping methods. First, we observed that the methods tested here suffer from a limitation of SV types that they are able to assess. None of the methods were able to assess novel insertions that also represent repeat expansions, which is a subclass of SVs recognized to for their impact in cancer and other phenotypes. Second, most of the methods suffer from a very strict VCF formatting requirement ignoring the current standard and relied further on individual flags that are hard or impossible to regenerate.

Overall STIX performed well on simulated and GiaB based SVs calls. It showed a good balance of sensitivity vs false discovery and was able to be run on standard VCF files. Nevertheless, the lack of genotype estimations is a clear limitation since it is often relevant to know if a variation is heterozygous or homozygous. Overall, the current methods, although limited in performance,

represents an advantage to diagnose patients with SVs compared to the discovery of SVs simply because of their much reduced false discovery rate.

Potential implications

SVs genotyping represents a possibility to infer SVs in clinical diagnosis by solving the problems of false discovery and false negative called SVs, compared to the discovery SV methods. However, genotyping SVs methods seem to require additional development to improve their ability to operate on different size regimes and all types of SVs (including insertions). Here we presented an overview of the current state-of-the-art methods, which highlights the need to improve upon the current state of the art to enable SV diagnosis of patients in clinical setups.

Methods

Simulated datasets

We simulated 20 SVs per dataset each for a certain type (indel, inversions, duplication and translocation) and a certain size (100bp, 250bp, 500bp, 1kbp, 2kbp, 5kbp, 10kbp, 50kbp) for chr 21 and 22 using SURVIVOR simSV. These simulations included a 1% SNP rate. After the simulation of the sample genomes we simulated reads using Mason [31] with the following parameter "Illumina -ll 500 -n 100 –N 39773784 -sq -mp -rn 2 " to generate 100bp paired-end Illumina like reads. The reads were mapped with BWA MEM[32] using the –M option to mark duplicated reads to the entire genome (GRCh38-2.1.0). Subsequently, we ran Manta (v1.2.1), DELLY (v0.7.8) and Lumpy (v0.2.13) to call SVs over the simulated datasets. For each data set we generated a union call set based on all 3 callers using SURVIVOR merge (v1.0.3) allowing

1kbp distance and allowing only the same SV type to be merged. This union set, as well as the SV genotyper output, was evaluated with SURVIVOR eval for the following categories: Precise: calling an SVs within 10bp and inferring the correct type. Indicated: allowing a maximum of 1kbp between the simulated and the called breakpoints and ignoring the predicted type of SVs. Missing: a simulated SVs but not re identified. Additional: a SVs that was called, but not simulated. The results were summarized using a costume R script operating on the output of SURVIVOR available on request.

SV genotyping: simulated data

For genotyping the simulated data set we used the union call VCF based on the SURVIVOR output as described above. We used DELLY (v0.7.8) specifying the output (-o), the vcf to be genotyped (-v) and the reference file (-g) as fasta and the bam file. We ran DELLY with the VCF file from SURVIVOR over the SV discovery caller. The obtained output from DELLY was converted using bcftools view (v1.7 (using htslib 1.7)) [26] to obtain a VCF file and was filtered to ignore genotyped calls with 0/0. SVTyper (v0.1.4) was used on the VCF generated from SURVIVOR based on the discovery phase. We filtered the obtained VCF for genotypes that could not have been accessed by SVTyper. SV2 (version 1.4.3) was run on the SURVIVOR generated VCF file for SVs genotyping but required also a SNV file. We generated this SNV file using Freebayes (v1.1.0-46-g8d2b3a0-dirty) [33] with the default parameters. The resulting SNV file from Freebayes was compressed and indexed by bgzip and tabix –p vcf [34], respectively. SV2 report their result in three folders (sv2_preprocessed, sv2_features and sv2_genotypes) from which we used the result reported in sv2_genotypes to benchmark the method. Genome

STRIP(v2.00.1774) was used following the suggested parameters and the VCF file generated by SURVIVOR. STIX (early version available over GitHub on April 6th 2018) was used to index the bam file using giggle (v0.6.3) [27], excord (v0.2.2) and samtools (v1.7) [26] following the suggested pipeline. Next, we run STIX with “-s 500” on the VCF files from SURVIVOR and ignoring output VCF entries with "STIX_ZERO=1", which filters out entries where STIX does not find any evidence for the SV.

SV genotyping: GiaB

We obtained the GiaB SV call set (v0.5.0) from ftp://ftp-trace.ncbi.nlm.nih.gov/giab/ftp/data/AshkenazimTrio/analysis/NIST_UnionSVs_12122017/ . The SNV calls were taken from here ftp://ftp-trace.ncbi.nlm.nih.gov/giab/ftp/release/AshkenazimTrio/HG002_NA24385_son/latest/GRCh37/ and the corresponding bam file from ftp://ftp-trace.ncbi.nlm.nih.gov/giab/ftp/data/AshkenazimTrio/HG002_NA24385_son/NIST_HiSeq_HG002_Homogeneity10953946/NHGRI_Illumina300X_AJtrio_novoalign_bams/HG002.hs37d5.300x.bam . The SVs call set needed to be filtered and reduced for just one sample (HG002) using cat and SURVIVOR and was subsequently filtered for deletions only. We ran all SV genotyping methods like described above. Subsequently,we filtered the results for genotypes: 0/1 and 1/1 with the exception of STIX. STIX was filtered base on if it reports reads to support the SVs or not. This was necessary since STIX does currently not report genotypes. After filtering we merged all data sets together including the original VCF provided using SURVIVOR with a maximum distance of 10bp and requiring the same types. We analyzed these merged calls

based on if the original call set reported a genotype to be heterozygous or homozygous alternative. The Venn diagram was generated based on the support vector reported by SURVIVOR and the R package Venn.diagram. The length of the SVs that were able to be genotyped were extracted using awk filtering for existing calls.

Availability of data and materials

The data sets used in this study are available here ftp://ftp-trace.ncbi.nlm.nih.gov/giab/ftp/data/AshkenazimTrio/analysis/NIST_UnionSVs_12122017/ and from ftp://ftp-trace.ncbi.nlm.nih.gov/giab/ftp/data/AshkenazimTrio/HG002_NA24385_son/NIST_HiSeq_HG002_Homogeneity10953946/NHGRI_Illumina300X_AJtrio_novoalign_bams/HG002.hs37d5.300x.bam. The simulated data sets are available on request.

Funding

This research was supported by National Institutes of Health award (UM1 HG008898).

Authors' contributions

VC and FS performed the analysis. VC, FS and RG wrote the manuscript. FS and RG directed the project.

Ethics approval and consent to participate

Not applicable

Consent for publication

Not applicable

Competing interests

F.J.S. has participated in PacBio sponsored meetings over the past few years and have received travel reimbursement and honoraria for presenting at these events

References

1. Weischenfeldt J, Symmons O, Spitz F and Korbel JO. Phenotypic impact of genomic structural variation: insights from and for human disease. Nat Rev Genet. 2013;14 2:125-38. doi:10.1038/nrg3373.
2. Lupski JR. Structural variation mutagenesis of the human genome: Impact on disease and evolution. Environ Mol Mutagen. 2015;56 5:419-36. doi:10.1002/em.21943.
3. Macintyre G, Ylstra B and Brenton JD. Sequencing Structural Variants in Cancer for Precision Therapeutics. Trends Genet. 2016;32 9:530-42. doi:10.1016/j.tig.2016.07.002.
4. Consortium GT, Laboratory DA, Coordinating Center -Analysis Working G, Statistical Methods groups-Analysis Working G, Enhancing Gg, Fund NIHC, et al. Genetic effects on gene expression across human tissues. Nature. 2017;550 7675:204-13. doi:10.1038/nature24277.

5. Jeffares DC, Jolly C, Hoti M, Speed D, Shaw L, Rallis C, et al. Transient structural variations have strong effects on quantitative traits and reproductive isolation in fission yeast. Nat Commun. 2017;8:14061. doi:10.1038/ncomms14061.

6. Sebat J, Lakshmi B, Troge J, Alexander J, Young J, Lundin P, et al. Large-scale copy number polymorphism in the human genome. Science. 2004;305 5683:525-8. doi:10.1126/science.1098918.

7. Sudmant PH, Rausch T, Gardner EJ, Handsaker RE, Abyzov A, Huddleston J, et al. An integrated map of structural variation in 2,504 human genomes. Nature. 2015;526 7571:75-81. doi:10.1038/nature15394.

8. Tattini L, D'Aurizio R and Magi A. Detection of Genomic Structural Variants from Next-Generation Sequencing Data. Front Bioeng Biotechnol. 2015;3:92. doi:10.3389/fbioe.2015.00092.

9. Alkan C, Coe BP and Eichler EE. Genome structural variation discovery and genotyping. Nat Rev Genet. 2011;12 5:363-76. doi:10.1038/nrg2958.

10. English AC, Salerno WJ and Reid JG. PBHoney: identifying genomic variants via long-read discordance and interrupted mapping. BMC Bioinformatics. 2014;15:180. doi:10.1186/1471-2105-15-180.

11. Mills RE, Walter K, Stewart C, Handsaker RE, Chen K, Alkan C, et al. Mapping copy number variation by population-scale genome sequencing. Nature. 2011;470 7332:59-65. doi:10.1038/nature09708.

12. Teo SM, Pawitan Y, Ku CS, Chia KS and Salim A. Statistical challenges associated with detecting copy number variations with next-generation sequencing. Bioinformatics. 2012;28 21:2711-8. doi:10.1093/bioinformatics/bts535.

13. Sedlazeck FJ, Rescheneder P, Smolka M, Fang H, Nattestad M, von Haeseler A, et al. Accurate detection of complex structural variations using single-molecule sequencing. Nat Methods. 2018; doi:10.1038/s41592-018-0001-7.

14. Goodwin S, McPherson JD and McCombie WR. Coming of age: ten years of next-generation sequencing technologies. Nat Rev Genet. 2016;17 6:333-51. doi:10.1038/nrg.2016.49.

15. Chaisson MJ, Huddleston J, Dennis MY, Sudmant PH, Malig M, Hormozdiari F, et al. Resolving the complexity of the human genome using single-molecule sequencing. Nature. 2015;517 7536:608-11. doi:10.1038/nature13907.

16. Merker JD, Wenger AM, Sneddon T, Grove M, Zappala Z, Fresard L, et al. Long-read genome sequencing identifies causal structural variation in a Mendelian disease. Genet Med. 2018;20 1:159-63. doi:10.1038/gim.2017.86.

17. Pedersen BS, Layer RM and Quinlan AR. Vcfanno: fast, flexible annotation of genetic variants. Genome Biol. 2016;17 1:118. doi:10.1186/s13059-016-0973-5.

18. Sedlazeck FJ, Dhroso A, Bodian DL, Paschall J, Hermes F and Zook JM. Tools for annotation and comparison of structural variation. F1000Res. 2017;6:1795. doi:10.12688/f1000research.12516.1.

19. Lappalainen I, Lopez J, Skipper L, Hefferon T, Spalding JD, Garner J, et al. DbVar and DGVa: public archives for genomic structural variation. Nucleic Acids Res. 2013;41 Database issue:D936-41. doi:10.1093/nar/gks1213.

20. Zook JM, Catoe D, McDaniel J, Vang L, Spies N, Sidow A, et al. Extensive sequencing of seven human genomes to characterize benchmark reference materials. Sci Data. 2016;3:160025. doi:10.1038/sdata.2016.25.

21. Rausch T, Zichner T, Schlattl A, Stutz AM, Benes V and Korbel JO. DELLY: structural variant discovery by integrated paired-end and split-read analysis. Bioinformatics. 2012;28 18:i333-i9. doi:10.1093/bioinformatics/bts378.

22. Handsaker RE, Van Doren V, Berman JR, Genovese G, Kashin S, Boettger LM, et al. Large multiallelic copy number variations in humans. Nat Genet. 2015;47 3:296-303. doi:10.1038/ng.3200.

23. Layer RM: https://github.com/ryanlayer/stix (2018).

24. Antaki D, Brandler WM and Sebat J. SV2: accurate structural variation genotyping and de novo mutation detection from whole genomes. Bioinformatics. 2018;34 10:1774-7. doi:10.1093/bioinformatics/btx813.

25. Chiang C, Layer RM, Faust GG, Lindberg MR, Rose DB, Garrison EP, et al. SpeedSeq: ultra-fast personal genome analysis and interpretation. Nat Methods. 2015;12 10:966-8. doi:10.1038/nmeth.3505.

26. Li H. A statistical framework for SNP calling, mutation discovery, association mapping and population genetical parameter estimation from sequencing data. Bioinformatics. 2011;27 21:2987-93. doi:10.1093/bioinformatics/btr509.

27. Layer RM, Pedersen BS, DiSera T, Marth GT, Gertz J and Quinlan AR. GIGGLE: a search engine for large-scale integrated genome analysis. Nat Methods. 2018;15 2:123-6. doi:10.1038/nmeth.4556.

28. McKenna A, Hanna M, Banks E, Sivachenko A, Cibulskis K, Kernytsky A, et al. The Genome Analysis Toolkit: a MapReduce framework for analyzing next-generation DNA sequencing data. Genome Res. 2010;20 9:1297-303. doi:10.1101/gr.107524.110.

29. Layer RM, Chiang C, Quinlan AR and Hall IM. LUMPY: a probabilistic framework for structural variant discovery. Genome Biol. 2014;15 6:R84. doi:10.1186/gb-2014-15-6-r84.

30. Chen X, Schulz-Trieglaff O, Shaw R, Barnes B, Schlesinger F, Kallberg M, et al. Manta: rapid detection of structural variants and indels for germline and cancer sequencing applications. Bioinformatics. 2016;32 8:1220-2. doi:10.1093/bioinformatics/btv710.

31. Holtgrewe M. *Mason-A Read Simulator for Second Generation Sequencing Data*. 2010. Institut für Mathematik und Informatik, Freie Universität Berlin.

32. Li H. Aligning sequence reads, clone sequences and assembly contigs with BWA-MEM. ArXiv e-prints. 2013; doi: arXiv:1303.3997 [q-bio.GN].

33. Garrison E and Marth G. Haplotype-based variant detection from short-read sequencing. ArXiv e-prints. 2012.

34. Li H. Tabix: fast retrieval of sequence features from generic TAB-delimited files. Bioinformatics. 2011;27 5:718-9. doi:10.1093/bioinformatics/btq671.

Click here to access/download
Supplementary Material
Tables.xlsx

Click here to download Personal Cover Coverletter.pdf

Baylor College of Medicine
Human Genome Sequencing Center

One Baylor Plaza
Houston, Texas 77030-3498

TEL: (713)798-6539
FAX: (713)798-5741

February 1, 2019

To the editors,

We are pleased to share our manuscript with you for consideration as a manuscript in GigaScience. Our work entitled "Evaluation of computational genotyping of Structural Variations for clinical diagnoses.", represents the first systematically assessment of Structural Variation (SV) genotyping methods and their individual advances and disadvantages.

As you may know, calling SVs using short reads is often discussed as error-prone and lacking of sensitivity. On the other hand, more and more papers appear highlighting the impact of SVs on different human diseases and across phenotypes among many species ranging from bacteria to humans. Here we suggest to utilize SV genotyping as a way to overcome this paradigm and to scan for annotated SVs in short read based sequencing data sets on a routine basis. This reduces the false positive rate significantly compared to short read based de novo SV calling.

In our manuscript, we further assess the ability and state of the art of these methods to recall SVs. This is first done over simulated data but then extended using the new gold standard from GIAB. Here we focus on variants that short read based calling is blind to and that were only found with long read technologies. We could show that our proposed strategy enables the detection of SVs also with short read based methods. This allows a broader screen for these important variant class for multiple studies and even a path forward for clinical screenings.

In the end, we conclude giving advice to which method performed the best and which method to choose to accomplish these goals. We further highlight general problems to the currently existing methods and make suggestions for future developments. Thus, we think our manuscript is of interest to the readers of GigaScience and in general for people that are currently interested in SVs and genetic diversity, but are cautious about the low performance of SV calling based on short read data.

We welcome any comments that you and your reviewers may have. We would recommend: Ryan Layer (ryan.layer@gmail.com), Aaron Quinland (aaronquinlan@gmail.com), Justin Zook (justin.zook@nist.gov) and Marc Salit (msalit@stanford.edu).

Sincerely,

Fritz J. Sedlazeck, Ph.D.

Assistant Professor,
Human Genome Sequencing Center,
Baylor College of Medicine,
Houston, TX, 77030
