# Supplementary material for: Evaluation of computational genotyping of structural variation for clinical diagnoses
Source: Gigascience. 2019 Sep 8;8(9):giz110. doi: 10.1093/gigascience/giz110 (PMC6732172; doi:10.1093/gigascience/giz110)
Supplement: giz110_GIGA-D-19-00035_Revision_1 [file giz110_giga-d-19-00035_revision_1.pdf]

GigaScience

Evaluation of computational genotyping of Structural Variations for clinical diagnoses

--Manuscript Draft--

**Manuscript Number:**	GIGA-D-19-00035R1
**Full Title:**	Evaluation of computational genotyping of Structural Variations for clinical diagnoses
**Article Type:**	Research
**Funding Information:**	National Human Genome Research Institute (UM1 HG008898) — Dr. Richard A. Gibbs
**Abstract:**	Background:<br>In recent years, Structural Variation (SV) has been identified as having a pivotal role in causing genetic disease. The discovery of SVs based on short DNA sequence reads from next-generation DNA sequence methods is error-prone, suffering from low sensitivity and high false discovery. These shortcomings can be partially overcome with extensive orthogonal validation methods, or use of long reads, but currently the cost of either precludes their application for routine clinical diagnostics. In contrast, SV genotyping of known sites of SV occurrence is relatively robust. Structural Variant genotyping therefore offers a cost-effective clinical diagnostic tool, with potentially few false positives and low occurrence of false negatives, even when applied to short-read DNA sequence data.<br><br>Results:<br>We assess five state- of-the- art SV genotyping software methods, applied to short read sequence data. The methods are characterized based on their ability to genotype different SV types, spanning different size ranges. Furthermore, we analyze their ability to parse different VCF file sub-formats and assess their reliance on specific metadata. We compare the SV genotyping methods across a range of simulated and real data including SVs that were not found with Illumina data alone. We assess sensitivity and the ability to filter initial false discovery calls.<br><br>Conclusion:<br>Our results indicate that, although SV genotyping software methods have superior performance to SV callers, there are limitations that suggest the need for further innovation.
**Corresponding Author:**	Fritz Sedlazeck<br><br>UNITED STATES
**Corresponding Author Secondary Information:**	
**Corresponding Author's Institution:**	
**Corresponding Author's Secondary Institution:**	
**First Author:**	Varuna Chander
**First Author Secondary Information:**	
**Order of Authors:**	Varuna Chander<br>Richard A. Gibbs<br>Fritz J. Sedlazeck
**Order of Authors Secondary Information:**	
**Response to Reviewers:**	A: We thank the reviewers for their helpful suggestions and for highlighting the importance of this first side-by-side assessment of SV genotyping software. We were able to incorporate all the suggestions made and performed the suggested analysis as requested. The modified text is highlighted in red in the manuscript.

Powered by Editorial Manager® and ProduXion Manager® from Aries Systems Corporation

Reviewer reports:

Reviewer #1: In this manuscript Chander and colleagues compare the performance of several tools that have been developed to assess the presence of a target set of structural variants in a new sample, given an aligned sequence file and VCF as input. The introduction describes the problem in sufficient detail. The authors conclude that none of the methods is clearly superior for correctly genotyping samples. Moreover, it appears that none of the methods can be endorsed as a strong overall performer, and attempting to combine the results of several tools in a voting approach may be unwise due to either lack of coverage of certain classes of SV, or requirements that VCFs be pre-processed using specific tools. Overall, the impression is of a field grappling with a difficult problem, with tools that are not yet ready for general use by non-specialists.

The manuscript is technically well-executed. The writing requires some proof-reading.

A: We thank the reviewer for his recommendations. Yes, we agree that the field is in an early stage, which asserts the importance of such benchmarks to reveal the current state and highlight what is missing.

Major comments:

1) Figures for the accuracy of SV calling are derived from high-quality but older citations that used low-pass sequencing that was more prevalent 6-8 years ago (e.g. Mills Nature 2011). More recent studies of SV (at least in cancer) use deeper short read sequencing (e.g. 30-100x depth). These methods are applied to non-homogenous cell populations where not all cells will harbor a given SV. The introduction would be improved if the authors commented briefly on 1) the trade-offs between higher sequencing depth, SV calling accuracy, and cost; and 2) the different applications of SV genotyping in a germline vs. tumor context. These factors may influence the utility of a given tool for different applications.

A: We agree with the reviewer that recent studies focus on higher sequencing depth. We did that for our simulation study (30x) and utilized even the full 300x data set for GIAB. However, we disagree that these are outdated methods. The methods we focused here are for SV genotyping and not for the initial SV discoveries. Other publications have already presented benchmarking of the latter (e.g. Kosugi et al. 2019, PMID: 31159850; Sedlazeck et. al. 2018 and Nattestad et. al 2018, PMID: 29713083 and 29954844).

2) Table 1 should distinguish between tools that are agnostic to the SV calling tool and those such as Delly or SVTyper that require a VCF generated using a specific SV method.

A: We thank the reviewer for this suggestion and have modified the table accordingly.

3) It's not clear what lines 125-126 mean, "given the nature of the data". Please be explicit about results were expected, why they were expected, and the degree to which the observed results conformed to those expectations.

A: We have clarified that by this statement- “We discovered only 17 false positive calls after the initial SV discovery. This low number of false positives is in contrast to reports from other studies. However, we are using here simulated data which does not take into account the complexities involved in regions of SVs and other sequencing biases. Interestingly, while this simulated data set represents an ideal case we still missed around 17.25% of the simulated SVs.
“ at line 133

4) The authors test whether the SV genotypers fail to call incorrectly genotyped SVs; this seems a distinct task from whether they correctly report the absence of a given SV when it is truly not present in the sample.

A: In this benchmark, we have assessed true positives (SVs that are present and should be re-found by the SV genotypers), false positives (SVs that are present in the input VCF but not present in the sample) and false negatives (SVs that are present in

Powered by Editorial Manager® and ProduXion Manager® from Aries Systems Corporation

the sample and in the input VCF but were not re – identified). The case where SVs are provided in the input VCF but are not supported in the sample is reflected as false positives test cases. The case where reads don't show any significant distortion in this region is trivial and thus was not explicitly assessed.

Reviewer #2: This study is designed to evaluate tools for the genotyping or validation of structural variant calls, with regard to their accuracy, applicability to types of structural variant, and usability. The authors make a strong case for the importance of this evaluation, due to the high false positive rates of most structural variant calling techniques that rely on short read sequencing technology and the utility of genotyping SVs, as well as counting alleles for population-level studies. SV genotypers were evaluated against a set of simulated SVs of different types, then against a set of SVs identified in a real sample through the use of many different and complementary technologies and methods by the GIAB consortium. The conclusion of this study is that while SV genotypers can be used to improve the accuracy of SV calls, they require considerable enhancement in usability and general applicability.

I like the simulation experiment, but the analysis needs to be improved. First, the figure. The axes are not labeled, and the colors are not described. The yellow and the orange are so similar, and the plots are so small that I didn't realize they were different colors until I zoomed way in. I kept getting lost in the description about which SVs are supported by which method.

A: We thank the reviewer for this suggestion. We have followed the guidelines for preparing the figures and used colors that are suggested for color blind people. The figure is meant to give a general trend showing in green/orange and red for the different performance abilities of the SV genotypers. The precise numbers are provided in Supplementary Tables.

It may be worth using some other visual cue (maybe another color) to indicated that a particular method does not work for a particular SV type, instead of just saying it genotypes 0% of the SVs. For example, SVTYPER supports BNDs but just misses all of them while GenomeSTRIP doesn't even try to genotype BNDs. That different is important and it would be helpful if it was clearer.

A: We discussed these limitations in the introduction of the manuscript and now this is also highlighted in Table 1. Figure 1 represents the ability to call certain SV types and sizes, from a standard VCF file. To clarify the presentation, we removed the BND assessment from Figure1 since none of the methods were able to successfully provide those genotypes. In the example of SVTyper, it cannot call BND events since it requires specific input data provided only by Lumpy. For other SV types we have included tags that we could reproduce (e.g. CIPOS, CIEND) in the VCF files to enable a comparison. In contrast, GenomeSTRIP indeed only focuses on DEL/ DUP events, which is similar to SV2.

In the description, since the overall rates are so dependent on the supported SV types, it may be worth reorganizing this section around SV types instead of going through each method and given a single rate (e.g., for DEL the method A was x%, B was y% and C doesn't do DEL).

A: We have made minor modifications to the text, since we were motivated to illustrate the overall combined performance of the methods. Figure 1 already illustrates the different advantages and disadvantages of the individual methods based on the different types and size regimes.

Question on the simulation experiment. Were the events all HET?

A: The SVs were all homozygous. We have clarified this in the manuscript.

Why only test the events that were detected? I get that in a non-simulated scenario you will only test the SVs that you detect, but it would be interesting to test how/if undetected SVs can be genotyped. This is a claim that has been made from long-read sequencing and it seems you can test it here too.

A: Thank you for raising this point. We had these tests included over the GIAB data set where a multitude of SV were only detectable using long reads. We highlighted the ability of SVgenotypers to identify these events. Furthermore, in the simulation we benchmarked the case where there were false SV calls and the ability of the SV genotypers to detect these.

On line 147, I don't think you meant "filter out falsely called SVs." That part is about true positives. The next paragraph is about filtering false positives.

A: We have modified the main text to clarify this point. This was one of the points we assessed in the benchmarks. We used standard SV callers (Delly, Lumpy and Manta) over a union set to obtain SV calls over each simulated data set. This also included a 17 falsely called SVs due to mapping errors or other reasons. We used these 17 artifacts to benchmark how these SV genotypers perform over a false indication of an SV in the sample. This could, for example, represent regions that are repetitive or otherwise challenging.

In the false positive part, you say that STIX does better than SVTYPER, but the numbers given do not seem to support that. STIX filters 76.47% and SVTYPER filters 81.82%. I am guessing the 81.82 is typo since you can't get to that number with 17 as a denominator.

A: We apologize for this confusion. The numbers reported in the Supplementary table were correct. We corrected this sentence: "… Genome STRiP performed best with filtering out all falsely detected SVs, but suffers from the lowest ability to genotype SV variation. STIX performed better as it can filter out 13 (76.4 %) of the false positive SV calls. In contrast, STIX also achieved a higher (71.76%) performance for correctly identifying SVs. Although SVTyper had the highest accurately genotyped SVs, it filtered out less of the false positives (69.70%) obtained during the discovery phase."

The dependence that some methods have on particular VCF flags is interesting, but I think you should comment on if either meet the VCF spec.

A: We clarified this in the main text. The issue is that all the input VCFs conform to the expected standard, but many tools require additional flags, which are not provided by other methods and are not easily reproducible.

This study, like most which deal with SV detection methods, suffered from a lack of fully reliable positive controls. The combination of simulated data and highly vetted GIAB SV calls provide a likely best currently possible answer to that problem. The low number of false positive SV calls in the simulated data suggest that the simulation was a best-case scenario for SV calling and therefore genotyping. Testing against a curated set of known false calls from previous published work might provide a useful complementary test of how well the genotypers handle false positives.

A: We appreciate the comment – and the recognition of the difficulty of providing a 'gold standard' for this kind of work. In the simulation, we focused on the SVs that are falsely called but are in regions that show mapping errors. The other cases, as suggested here, would be exemplified by an SV in an input VCF vs. non-altered mapping within specified regions. These cases are easily distinguishable by e.g. a lack of abnormally mapped reads and thus we did not assess this.

Use of the GIAB SV callset as a second test case for the genotypers is a valuable exercise and demonstrates the performance of these genotypers in real data. A mostly unavoidable source of concern is the reliability of the calls from GIAB that are used in this experiment. These calls are an attempt to sensitively identify all structural variation in the Ashkenazi Son sample and seem likely (due to the number of events) to contain a large number of false positives. This could be reflected in the number of variants that were not detected by any of the genotypers, but those could also represent real variants that genotypers could not identify. It would therefore strengthen the argument to have some additional analysis of the variants that were not identified by any genotyper, such as a downsampling and visual review. If the majority of those variants appear to be false positives in GIAB rather than false negatives in genotyping, the

Powered by Editorial Manager® and ProduXion Manager® from Aries Systems Corporation

	performance of the genotypers may potentially be much stronger than it currently appears to be.<br><br>A: We agree with the reviewer that this could have been a potential pitfall. However, the GIAB calls in v0.5 have been produced via multiple rounds of manual curation using various sequencing technologies and assembly and mapping approaches. The combined high confidence set within the high confidence regions indeed represents a highly accurate SV call set that has been assessed multiple times by us and others over various studies. Hence, we do not share this concern.<br><br>How dependent is the performance of STIX on finding just one read supporting an SV?<br><br>A: As we highlighted in the main text, it is a disadvantage of STIX to only report read counts vs. genotypes from other methods. Since this is a limitation of the method itself, we can just highlight this in the discussion as we did.<br><br>A missing piece for all of the experiments is runtime. Is one of these more efficient than the others?<br><br>A: Thank you for this suggestion. We have included the average CPU time measured over 20 runs as Supplementary Table. STIX is the fastest method (0.4 seconds) followed by Delly (3.7s) and SVtyper (9.6s). The slowest by far is GenomeSTRiP (33.8 min).
**Additional Information:**	
**Question**	**Response**
Are you submitting this manuscript to a special series or article collection?	No
**Experimental design and statistics**<br><br>Full details of the experimental design and statistical methods used should be given in the Methods section, as detailed in our Minimum Standards Reporting Checklist. Information essential to interpreting the data presented should be made available in the figure legends.<br><br>Have you included all the information requested in your manuscript?	Yes
**Resources**<br><br>A description of all resources used, including antibodies, cell lines, animals and software tools, with enough information to allow them to be uniquely identified, should be included in the Methods section. Authors are strongly encouraged to cite Research Resource Identifiers (RRIDs) for antibodies, model organisms and tools, where possible.	Yes

Have you included the information requested as detailed in our Minimum Standards Reporting Checklist?	
**Availability of data and materials**<br><br>All datasets and code on which the conclusions of the paper rely must be either included in your submission or deposited in publicly available repositories (where available and ethically appropriate), referencing such data using a unique identifier in the references and in the “Availability of Data and Materials” section of your manuscript.<br><br>Have you have met the above requirement as detailed in our Minimum Standards Reporting Checklist?	Yes

Manuscript
Click here to access/download;Manuscript;GIGA-D-19-00035_Manuscript.docx
Click here to view linked References

Evaluation of computational genotyping of Structural Variations for clinical diagnoses.

Varuna Chander[1], Richard A. Gibbs[1], Fritz J. Sedlazeck[1]*

1: Human Genome Sequencing Center, Baylor College of Medicine, One Baylor Plaza, Houston TX 77030

VC: Varuna.Chander@bcm.edu

RG: agibbs@bcm.edu

FJS: fritz.sedlazeck@bcm.edu

* Correspondence: fritz.sedlazeck@bcm.edu

Abstract

Background:

In recent years, Structural Variation (SV) has been identified as having a pivotal role in causing genetic disease. The discovery of SVs based on short DNA sequence reads from next-generation DNA sequence methods is error-prone, suffering from low sensitivity and high false discovery. These shortcomings can be partially overcome with extensive orthogonal validation methods, or use of long reads, but currently the cost of either precludes their application for routine clinical diagnostics. In contrast, SV genotyping of known sites of SV occurrence is relatively robust. Structural Variant genotyping therefore offers a cost-effective clinical diagnostic tool, with potentially few false positives and low occurrence of false negatives, even when applied to short-read DNA sequence data.

Results:

We assess five state- of-the- art SV genotyping software methods, applied to short read sequence data. The methods are characterized based on their ability to genotype different SV types, spanning different size ranges. Furthermore, we analyze their ability to parse different VCF file sub-formats and assess their reliance on specific metadata. We compare the SV genotyping methods across a range of simulated and real data including SVs that were not found with Illumina data alone. We assess sensitivity and the ability to filter initial false discovery calls.

Conclusion:

Our results indicate that, although SV genotyping software methods have superior performance to SV callers, there are limitations that suggest the need for further innovation.

Keywords (3-10)

Structural Variations, Genotyping, clinical diagnosis, Next Generation Sequencing

Background

With the continuous advancement of sequencing technologies, our understanding of the importance of Structural Variation (SV) is increasing[1]. Structural Variation has a critical role in evolution[2], genetic diseases (e.g. mendelian or cancer) [2, 3] and the regulation of genes in

different cells and tissues[4]. Furthermore, SVs constitute a substantial proportion of the genomic differences between cell types, individuals, populations and species [1, 4-8]. Structural Variation is generally defined as 50bp or longer genomic variation and is categorized into five types: Insertions, Deletions, Duplications, Inversions and Translocations [9]. Structural Variation is most often identified by leveraging combinations of paired-end, split read signals and coverage information[8].

Methods for the *de novo* detection of SVs are still in their infancy, with some procedures reporting high (up to 89%) levels of false discovery[7, 8, 10-12] (i.e. SVs that are inferred due to artifacts, but not truly present in the sample) and between 10% to 70% false negatives[5, 7] (i.e. missing present SVs in the samples). Although deeper DNA sequence coverage is often used to improve *de novo* discovery of SVs, for example in cancer samples [13], this alone does not solve the sensitivity and accuracy shortcomings. The performance of these methods can be improved by the use of long DNA sequence reads, however this is often not practical due to high sequencing costs [14-16]. Therefore, using short reads alone significantly hinders SV discovery for routine clinical diagnosis [17].

An additional challenge is the interpretation of the possible functional consequences of SVs. Despite the availability of existing methods to compare SVs (e.g. SURVIVOR [5]) and to study the potential impact of SVs on genes (VCFanno [18], SURVIVOR_ant [19]), there is still a paucity of methods to assess their allele frequency among human populations. These issues can hinder

routine screening for SVs and limit their proper recognition and characterization for clinical diagnoses.

The identification of SVs that have been previously identified in different samples is, in principle, easier than *de novo* detection. For known SVs it is possible to computationally detect SVs directly from short read DNA sequence data in data from individual patient samples, guided by the expected position of split reads and discordant paired reads that can confirm breakpoints. This less demanding approach reduces false discovery rates and therefore renders the methods more suitable for clinical applications. In addition, the false negative rate can be reduced as it is easier to genotype a variant than to identify a new SV. Focusing on known SVs has further the advantage, compared with *de novo* discovery of SVs, that SV databases will have likely recorded the event, together with its possible association with disease (e.g. dbVar [20]).

Here, we review the current state of SVs genotyping methods and investigate their potential for application in clinical diagnoses. In particular, we address whether these SV calling softwares (‘ SV genotypers’) can re-identify SVs that short read *de novo* SV callers failed to identify (over GIAB [21, 22]call sets) and how they perform on initially falsely inferred SVs. We describe which SV genotypers most efficiently identify which types of SVs and the effect of SV sizes.

Analyses

Existing methods

We assessed SVs genotypers: DELLY [23], Genome STRiP [24], STIX[25], SV2 [26] and SVTyper [27]. They share a common feature in which they require a bam file of the mapped reads and a VCF file that will be genotyped for SVs as inputs. **Table 1** lists their dependencies and their ability to genotype certain types of SVs.

Overall, they can be divided into groups that support only two SV types (e.g. Genome STRiP) up to methods that support all SV types (SVTyper and DELLY), but require specific meta-information to do so. In the following, we give a brief description of each method that we assessed. Further insights can be obtained from their respective publications or manuals.

Genotyper	Approach	SV Type					Inputs	Dependencies
		Del	Ins	Inv	Dup	TRA/BND		
**Delly**	RD, PR, SR	✓		*	*	*	BAM, VCF, Ref	Bcftools [28]
**Svtyper**	SR, PR	✓		✓	✓	*	BAM, VCF, Ref	
**SV2**	RD, PR, SR	✓			✓		BAM, SNV VCF, VCF, Ref, PED file	
**STIX**	PR,SR	✓		✓			BAM compressed, PED file, VCF, Ref	Excord, Giggle [29]

**Genome StRiP**	RD, PR, SR	✓			✓		BAM, VCF, Ref	GATK[30]

*Table 1: Overview of the SV genotypers assessed here and their ability to assess different SV types. ✓ : works on a standardized VCF file. *: marks dependencies on specialized tags in the VCF files. RD: read depth, SR: split reads, PR: paired end reads*

DELLY[23] is originally an SV caller that includes a genotype mode to redefine multi-sample VCFs. It operates on split and paired-end reads to genotype deletions, duplications, inversions and translocations. However, for all types except the deletions, DELLY requires a sequence resolved call in its own format to be able to estimate the genotype.

Genome STRiP[24] genotypes only deletions and duplications. The unique aspect of Genome STRiP is that it was designed to genotype multiple samples simultaneously. It requires the GATK pipeline and prepackaged reference metadata bundles.

STIX[25], which is the most recently developed method included here, utilizes a reverse approach to the previous two examples. First, STIX extracts the discordant read pairs and split reads and generates a searchable index per sample. This index can then be queried if it supports a specific variant call. Noteworthy, STIX in the current form only provides information on how many reads support a variant rather than the genotype itself. This is done with a flag describing whether the reads are supported by a particular variant and the number of reads supporting it.

SVTyper[27] uses a Bayesian likelihood model that is based on discordant paired-end reads and split reads. It was designed to genotype deletions, duplications, inversions and translocations. For the latter, however, SVTyper requires specific ID tags provided by Lumpy[31] to complete genotyping.

SV2[26] uses a support vector machine learning to genotype deletions and duplications based on discordant paired-end, split read and coverage. Furthermore, it was the only SV genotyper assessed here that leverages SNP calls for its prediction.

Evaluation of SVs computational genotypers based on simulated data

To first assess the performance of genotyping methods for SVs, we simulated data sets with 100bp Illumina like paired-end reads. Each data set includes 20 homozygous SVs simulated for a certain SVs type (duplications, indels, inversions and translocation) and a certain size range (100bp, 250bp, 500bp, 1kbp, 2kbp, 5kbp, 10kbp, 50kbp). For each of the data sets, we called SVs using SURVIVOR[5] based on a union set of DELLY, Manta[32], Lumpy[31] calls to include true positive as well as false positive SVs calls (see methods).

We discovered only 17 false positive calls after the initial SV discovery. This low number of false positives is in contrast to reports from other studies. However, we are using here simulated data which does not take into account the complexities involved in regions of SVs and other sequencing biases. Interestingly, while this simulated data set represents an ideal case, we still missed around 17.25% of the simulated SVs.

Supplementary Table 1 shows the results for the SV discovery set over the 32 simulated data sets based on 640 simulated SVs on chr21 and chr22.

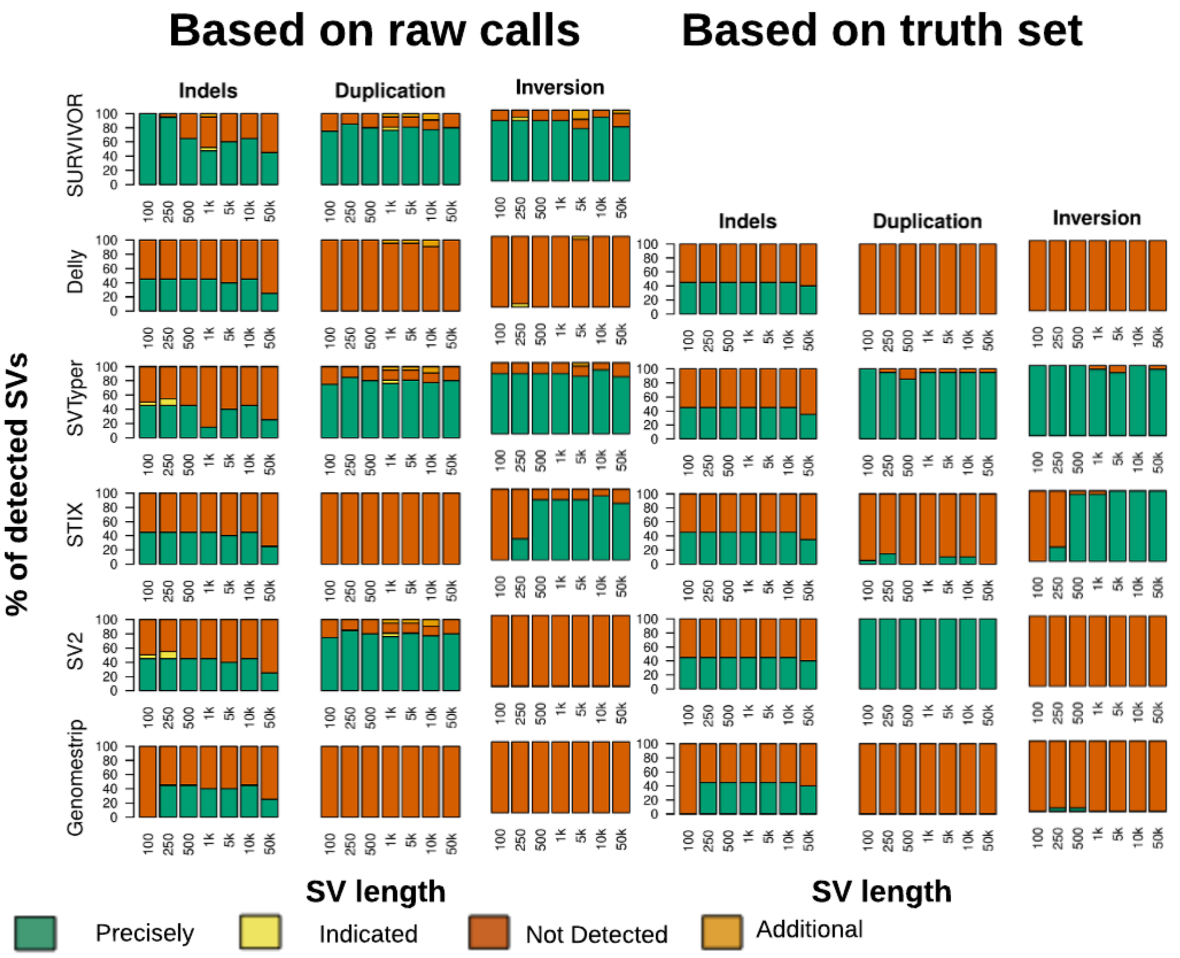

Figure 1: *Evaluation of Illumina like reads to assess the SV genotyper ability to re-identify insertions, deletions, duplications and inversions over different size ranges (x-axis). The colors indicate the SVs being detected/ genotyped by the respective SV genotypers. They were classified either precisely (green), indicated (yellow), not detected (red) or falsely identified (brown) (see Methods). For the SVs genotyped based on SV calls (left) we used SURVIVOR is a union set of Delly, Lumpy and Manta to generate the VCF file as an input for the SV genotypers. Noteworthy, Delly and SVtyper can genotype more SVs, given the custom information from their respective callers- Delly and SVTyper, respectively. When the truth SV set is provided as a start point (right side) we see marginal improvements across the SV genotyping methods while maintaining the overall trend.*

The generated VCF files were taken as input for the five SV genotyper callers: DELLY, Genome STRiP, SV2, STIX and SVTyper. **Figure 1** provides an overview with respect to the ability to discover SVs in the first place (SURVIVOR). We did not visualize translocations/ BND since none of the genotypers were able to identify them based upon our standard conform VCF file. **Supplementary Table 1** shows the result for all SV genotypers, applied to the 32 simulated data sets.

Interestingly, we observed that certain methods require a specialized VCF file with information provided specific to a one SV caller. For example, while SVTyper is able to genotype deletions, inversion and duplications, it will work on BND (translocation) events only if the ID pairs provided by Lumpy are included in the VCF file. Additionally, DELLY, which is capable to infer deletions, inversion, duplications and translocations types of SVs is only able to genotype deletions given a standardized VCF is provided without the extra information.

The overall performance of each method was evaluated based on the input VCF generated by SURVIVOR. Thus, if all of the short-read based SV callers were not able to resolve the insertions of 5kbp, then it would be assessed as a 'wrong/missed' SV.

First, we assessed the ability of the SV genotypers to correctly genotype SVs. SVTyper (64.70%) had the highest rate of correctly genotyping SVs to be present, followed by SV2 (41.57%). Importantly, SV2 was able to genotype deletions and duplications, while SVTyper assessed deletions, duplications and inversions. Genome STRiP had the lowest (14.40%) success rate of

all methods because it can only genotype deletions and duplications. This result was expected considering Genome STRiP was designed primarily for population-based genotyping. SVTyper improved marginally (86.26%) when BND events, which represented translocations, were ignored, followed by the next best method SV2 (83.15%) when focused on deletions and duplications. Furthermore, we also benchmarked the SV genotyping methods on their performance, given the truth set (**Supplementary Table 2**). The different methods show performance differences in the runtime ranging from 0.3 seconds (STIX) to 33.8 minutes (GenomeSTRIP) (**Supplementary Table 3**).

Next, we assessed the ability of the SV genotypers to reduce the rate at which false positives were observed, i.e. initially wrongly inferred SVs. This represents the scenario of accidentally genotyping a SV that is not represented in the sample due to sequencing or mapping biases. Over the 32 call sets, SURVIVOR had only 17 false positive calls for the simulated data. Genome STRiP performed best with filtering out all falsely detected SVs, but suffered with the lowest ability to genotype SV variations. STIX performed better as it can filter out 13 (76.4%) of the false positive SV calls. In contrast, STIX also achieved a higher (71.76%) performance for correctly identifying SVs. Although SVTyper had the highest accurately genotyped SVs, it filtered out less of the false positives (70.59%) obtained during the discovery phase.

In summary, we observed that none of the methods were clearly superior for correctly genotyping and correctly filtering/non-reporting SV variation. Strikingly, none of the programs were able to genotype insertions or translocations in the simulated data sets. Nevertheless,

STIX and SV2 showed strong performance, with a good balance of sensitivity and being able to correctly discard false positives.

Evaluation of SVs computational genotypers based on GIAB Ashkenazy Son

We further assessed genotyping of SVs calls based on the long-read DNA sequence data from an 'Ashkenazi Son' (HG002) reference sample. Specifically, we tested the currently released call set (v0.5.0) from GIAB, generated using sequence resolved calls from multiple technologies such as Illumina, PacBio, BioNano etc. and multiple SV callers and *de novo* assemblies based on these technologies, alone or in combination [21]. It is important to note that 8,195 of these SV calls could not be initially discovered with any Illumina assembly or caller but originated from PacBio based calls or BioNano based calling.

We next utilized this call set to genotype the SVs based on a 300x Illumina bam file for HG002 and compare the obtained SV genotype predictions to the genotypes reported by GIAB. The first observation was that most of the SV genotypers were unable to process the VCF file provided by GIAB. We used SURVIVOR to reduce the information included in the GIAB VCF file. Next, we filtered out the reported INS and complex events from this call set as most SV genotypers failed computationally to complete assessing these entries. Unfortunately, we were not able to run GenomeSTRiP successfully as it repeatedly failed, even when applied to just a subset of these calls.

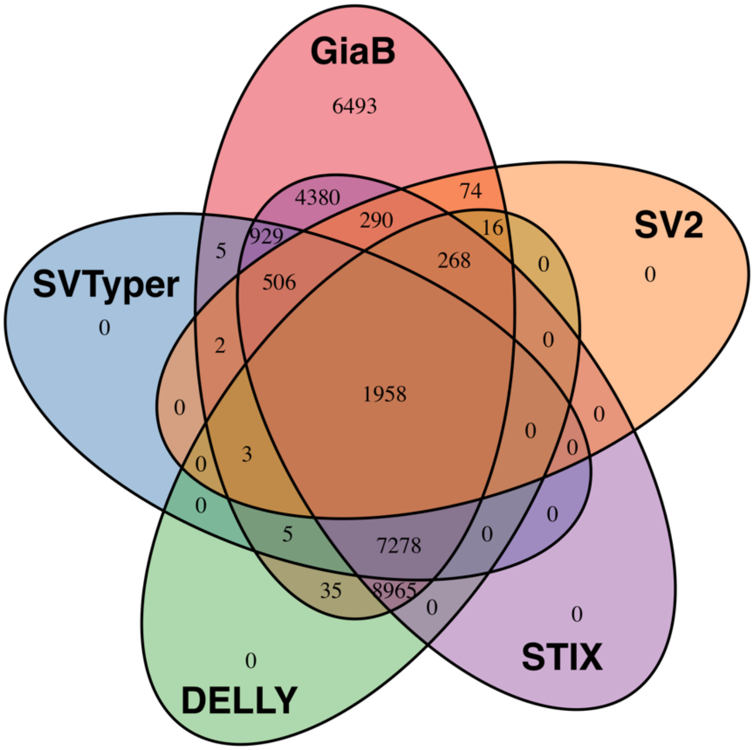

Figure 2: Evaluation based on GIAB call set v0.5.0 deletions only.

Figure 2 displays the detectable deletions based on the GIAB call set (v0.5.0) per SV genotyper. STIX performed the best among all methods identifying 24,574 (78.74%) of the provided deletions. It is important to note that STIX does not currently report genotypes. Thus, we relied only on the information if STIX found reads that support this event rather than genotype information. DELLY performed as the second best identifying 18,528 (59.37%) deletions followed by SVTyper (34.24%) and SV2 (9.99%). Only 6.27% of the deletion calls from GIAB call set were genotyped by all SV genotype methods. Although this is a very low percent, it is positive that up to 78.74% of the deletions could be successfully identified out of 62,676 deletions (20bp+) in total. Noteworthy, 4921 deletions out of this set were never observed by

any Illumina based caller or assembly. This highlights the potential benefit of using SV genotypers.

Next, we assessed the size ranges that SVs genotypers were able to recognize SVs. The deletions from GIAB call set 0.5.0 ranged from 20bp up to 997kbp with a median size of 36bp. All of the SV genotypers were able to identify deletions down to a size of 20bp. Interestingly we observed different median sizes of genotyped deletions, which represents the ability of specific methods to resolve small versus large events. DELLY (31bp) had the lowest median SV size followed by SVTyper (32bp), STIX (35bp) and SV2 (116bp). Furthermore, DELLY (816kbp) genotyped also the longest SVs followed by STIX (694kbp), SV2 (656bkbp) and SVTyper (656bkbp). See **Supplementary Table 4** for details.

When assessing the genotype concordance (see **Supplementary Table 5**), DELLY performed the best with an agreement rate of 87.08% given that it identified the variant in the first place. SV2 achieved a 78.59% of genotype agreement, however it had one of the lowest recall rates (9.99%). SVTyper showed a 67.79% genotypes concordance. We did not evaluate STIX in this perspective since it does not report a genotype estimation in its current version.

In summary, STIX and DELLY performed the best in re-identifying the deletions reported by GIAB for HG0002. Furthermore, DELLY (87.08%) had also the highest agreement over the genotypes with the GIAB call set.

Discussion

In this paper, we assessed the current state of SV genotyping methods. These methods are valuable for identifying the genotype of SVs in new samples, at sites of already known validated and functionally annotated SVs. The methods are important for diagnostic applications and as they offer better accuracy and reproducibility for the clinic than *de novo* detection methods.

A significant observation was that as a practical matter, many SV genotypers are limited to applications linked to their *de novo* SV caller counterpart. For example, DELLY successfully genotyped all SV types subsequent to its use as a discovery method, but only when supplied with the DELLY-specific VCF file. Similarly, SVTyper relies on specific IDs associated to translocations (in this case BND) events provided by Lumpy.

We provided the first assessments of sensitivity and false discovery rate for SV genotypers that include not only Illumina detectable SVs, but those that could only be initially discovered via long read technologies such as PacBio or Oxford Nanopore [14, 16]. These technologies often enable the detection of more complex SVs and those within regions that are difficult to resolve by Illumina alone – but are neither scalable or accurate enough to support routine *de novo* SV identification in a clinical setting [17].

This study also identified both general and method-specific limitations of SV genotyping methods. First, we observed that none of the methods tested were able to assess novel insertions that also represent repeat expansions, which is a subclass of SVs recognized as

important in cancer and other diseases. Second, most of the methods suffer from strict VCF formatting requirements, ignoring the current standards conventions, relying on individual flags that are difficult to emulate.

Among the SV genotypers, STIX performed best when applied to simulated and GIAB based SVs calls, demonstrating a good balance of high sensitivity versus reduced false discovery with the added ability to use standard VCF files. Nevertheless, the lack of genotype estimations for STIX remains a limitation. In aggregate, our results indicate SV genotypers have better performance than SV callers. Our approach can be integrated into existing analysis pipelines for routine scanning of known pathogenic SVs, representing an efficient and quick way to diagnose patients with SVs in the clinic.

Potential implications

SVs genotyping represents an opportunity to infer SVs in clinical diagnostic settings where low false discovery and false negative rates are critical. However, genotyping SVs methods seem to require additional development to improve their ability to operate on different size events and on all types of SVs (including insertions). Here we presented an overview of the current state-of-the-art methods, and highlight the need for specific methodological improvements.

Methods

Simulated datasets

We simulated 20 SVs per dataset each for a certain type (indel, inversions, duplication and translocation) and a certain size (100bp, 250bp, 500bp, 1kbp, 2kbp, 5kbp, 10kbp, 50kbp) for chr 21 and 22 using SURVIVOR simSV. These simulations included a 1% SNP rate. After the simulation of the sample genomes we simulated reads using Mason [33] with the following parameter “Illumina -ll 500 -n 100 –N 39773784 -sq -mp -rn 2 “ to generate 100bp paired-end Illumina like reads. The reads were mapped with BWA MEM[34] using the –M option to mark duplicated reads to the entire genome (GRCh38-2.1.0). Subsequently, we ran Manta (v1.2.1), DELLY (v0.7.8) and Lumpy (v0.2.13) to call SVs over the simulated datasets. For each data set we generated a union call set based on all 3 callers using SURVIVOR merge (v1.0.3) allowing 1kbp distance and allowing only the same SV type to be merged. To assess the performance of the SV genotypers across the truth set of SV, we used the output of SURVIVOR that is also used for the evaluation. Subsequently we converted that output to a VCF file using SURVIVOR bed2vcf. We incorporated CPOS and CIEND with both 0,0 to enable running SVTyper.

This union set, as well as the SV genotyper output, was evaluated with SURVIVOR eval for the following categories:

Precise: calling an SVs within 10bp and inferring the correct type. Indicated: allowing a maximum of 1kbp between the simulated and the called breakpoints and ignoring the predicted type of SVs. Missing: a simulated SVs but not re identified. Additional: a SVs that was called, but not simulated. The results were summarized using a custom R script operating on the output of SURVIVOR available on request.

The runtime of each method was measured across all simulated data set using Linux time and the average CPU time was reported.

SV genotyping: simulated data

For genotyping the simulated data set, we used the union call VCF based on the SURVIVOR output as described above. We used DELLY (v0.7.8) specifying the output (-o), the vcf to be genotyped (-v) and the reference file (-g) as fasta and the bam file. We ran DELLY with the VCF file from SURVIVOR over the SV discovery caller. The obtained output from DELLY was converted using bcftools view (v1.7 (using htslib 1.7)) [28] to obtain a VCF file and was filtered to ignore genotyped calls with 0/0. SVTyper (v0.1.4) was used on the VCF generated from SURVIVOR based on the discovery phase. We filtered the obtained VCF for genotypes that could not have been accessed by SVTyper. SV2 (version 1.4.3) was run on the SURVIVOR generated VCF file for SVs genotyping but required also a SNV file. We generated this SNV file using Freebayes (v1.1.0-46-g8d2b3a0-dirty) [35] with the default parameters. The resulting SNV file from Freebayes was compressed and indexed by bgzip and tabix –p vcf [36], respectively. SV2 report their result in three folders (sv2_preprocessed, sv2_features and sv2_genotypes) from which we used the result reported in sv2_genotypes to benchmark the method. Genome STRIP(v2.00.1774) was used following the suggested parameters and the VCF file generated by SURVIVOR. STIX (early version available over GitHub on April 6th 2018) was used to index the bam file using giggle (v0.6.3) [29], excord (v0.2.2) and samtools (v1.7) [28] following the suggested pipeline. Next, we run STIX with “-s 500” on the VCF files from SURVIVOR and

ignoring output VCF entries with "STIX_ZERO=1", which filters out entries where STIX does not find any evidence for the SV.

SV genotyping: GIAB

We obtained the GIAB SV call set (v0.5.0) from ftp://ftp-trace.ncbi.nlm.nih.gov/giab/ftp/data/AshkenazimTrio/analysis/NIST_UnionSVs_12122017/ . The SNV calls were taken from here ftp://ftp-trace.ncbi.nlm.nih.gov/giab/ftp/release/AshkenazimTrio/HG002_NA24385_son/latest/GRCh37/ and the corresponding bam file from ftp://ftp-trace.ncbi.nlm.nih.gov/giab/ftp/data/AshkenazimTrio/HG002_NA24385_son/NIST_HiSeq_HG002_Homogeneity10953946/NHGRI_Illumina300X_AJtrio_novoalign_bams/HG002.hs37d5.300x.bam . The SVs call set needed to be filtered and reduced for just one sample (HG002) using cat and SURVIVOR and was subsequently filtered for deletions only. We ran all SV genotyping methods like described above. Subsequently, we filtered the results for genotypes: 0/1 and 1/1 with the exception of STIX. STIX was filtered based on if it reports reads to support the SVs or not. This was necessary since STIX does currently not report genotypes. After filtering we merged all data sets together including the original VCF provided using SURVIVOR with a maximum distance of 10bp and requiring the same SV types. We analyzed these merged calls based on if the original call set reported a genotype to be heterozygous or homozygous alternative. The Venn diagram was generated based on the support vector reported by SURVIVOR and the R package Venn.diagram. The length of the SVs that were able to be genotyped were extracted using awk filtering for existing calls.

Availability of data and materials

The data sets used in this study are available here ftp://ftp-trace.ncbi.nlm.nih.gov/giab/ftp/data/AshkenazimTrio/analysis/NIST_UnionSVs_12122017/ and from ftp://ftp-trace.ncbi.nlm.nih.gov/giab/ftp/data/AshkenazimTrio/HG002_NA24385_son/NIST_HiSeq_HG002_Homogeneity10953946/NHGRI_Illumina300X_AJtrio_novoalign_bams/HG002.hs37d5.300x.bam. The simulated data sets are available on request.

Declarations

Funding

This research was supported by National Institutes of Health award (UM1 HG008898).

Authors' contributions

VC and FS performed the analysis. VC, FS and RG wrote the manuscript. FS and RG directed the project.

Ethics approval and consent to participate

Not applicable

Consent for publication

Not applicable

Competing interests

F.J.S. has participated in PacBio and Oxford Nanopore sponsored meetings over the past few years and have received travel reimbursement and honoraria for presenting at these events.

References

1. Weischenfeldt J, Symmons O, Spitz F and Korbel JO. Phenotypic impact of genomic structural variation: insights from and for human disease. Nat Rev Genet. 2013;14 2:125-38. doi:10.1038/nrg3373.
2. Lupski JR. Structural variation mutagenesis of the human genome: Impact on disease and evolution. Environ Mol Mutagen. 2015;56 5:419-36. doi:10.1002/em.21943.
3. Macintyre G, Ylstra B and Brenton JD. Sequencing Structural Variants in Cancer for Precision Therapeutics. Trends Genet. 2016;32 9:530-42. doi:10.1016/j.tig.2016.07.002.
4. Consortium GT, Laboratory DA, Coordinating Center -Analysis Working G, Statistical Methods groups-Analysis Working G, Enhancing Gg, Fund NIHC, et al. Genetic effects on gene expression across human tissues. Nature. 2017;550 7675:204-13. doi:10.1038/nature24277.

5. Jeffares DC, Jolly C, Hoti M, Speed D, Shaw L, Rallis C, et al. Transient structural variations have strong effects on quantitative traits and reproductive isolation in fission yeast. Nat Commun. 2017;8:14061. doi:10.1038/ncomms14061.

6. Sebat J, Lakshmi B, Troge J, Alexander J, Young J, Lundin P, et al. Large-scale copy number polymorphism in the human genome. Science. 2004;305 5683:525-8. doi:10.1126/science.1098918.

7. Sudmant PH, Rausch T, Gardner EJ, Handsaker RE, Abyzov A, Huddleston J, et al. An integrated map of structural variation in 2,504 human genomes. Nature. 2015;526 7571:75-81. doi:10.1038/nature15394.

8. Tattini L, D'Aurizio R and Magi A. Detection of Genomic Structural Variants from Next-Generation Sequencing Data. Front Bioeng Biotechnol. 2015;3:92. doi:10.3389/fbioe.2015.00092.

9. Alkan C, Coe BP and Eichler EE. Genome structural variation discovery and genotyping. Nat Rev Genet. 2011;12 5:363-76. doi:10.1038/nrg2958.

10. English AC, Salerno WJ and Reid JG. PBHoney: identifying genomic variants via long-read discordance and interrupted mapping. BMC Bioinformatics. 2014;15:180. doi:10.1186/1471-2105-15-180.

11. Mills RE, Walter K, Stewart C, Handsaker RE, Chen K, Alkan C, et al. Mapping copy number variation by population-scale genome sequencing. Nature. 2011;470 7332:59-65. doi:10.1038/nature09708.

12. Teo SM, Pawitan Y, Ku CS, Chia KS and Salim A. Statistical challenges associated with detecting copy number variations with next-generation sequencing. Bioinformatics. 2012;28 21:2711-8. doi:10.1093/bioinformatics/bts535.

13. Nattestad M, Goodwin S, Ng K, Baslan T, Sedlazeck FJ, Rescheneder P, et al. Complex rearrangements and oncogene amplifications revealed by long-read DNA and RNA sequencing of a breast cancer cell line. Genome Res. 2018;28 8:1126-35. doi:10.1101/gr.231100.117.

14. Sedlazeck FJ, Rescheneder P, Smolka M, Fang H, Nattestad M, von Haeseler A, et al. Accurate detection of complex structural variations using single-molecule sequencing. Nat Methods. 2018; doi:10.1038/s41592-018-0001-7.

15. Goodwin S, McPherson JD and McCombie WR. Coming of age: ten years of next-generation sequencing technologies. Nat Rev Genet. 2016;17 6:333-51. doi:10.1038/nrg.2016.49.

16. Chaisson MJ, Huddleston J, Dennis MY, Sudmant PH, Malig M, Hormozdiari F, et al. Resolving the complexity of the human genome using single-molecule sequencing. Nature. 2015;517 7536:608-11. doi:10.1038/nature13907.

17. Merker JD, Wenger AM, Sneddon T, Grove M, Zappala Z, Fresard L, et al. Long-read genome sequencing identifies causal structural variation in a Mendelian disease. Genet Med. 2018;20 1:159-63. doi:10.1038/gim.2017.86.

18. Pedersen BS, Layer RM and Quinlan AR. Vcfanno: fast, flexible annotation of genetic variants. Genome Biol. 2016;17 1:118. doi:10.1186/s13059-016-0973-5.

19. Sedlazeck FJ, Dhroso A, Bodian DL, Paschall J, Hermes F and Zook JM. Tools for annotation and comparison of structural variation. F1000Res. 2017;6:1795. doi:10.12688/f1000research.12516.1.

20. Lappalainen I, Lopez J, Skipper L, Hefferon T, Spalding JD, Garner J, et al. DbVar and DGVa: public archives for genomic structural variation. Nucleic Acids Res. 2013;41 Database issue:D936-41. doi:10.1093/nar/gks1213.

21. Zook JM, Catoe D, McDaniel J, Vang L, Spies N, Sidow A, et al. Extensive sequencing of seven human genomes to characterize benchmark reference materials. Sci Data. 2016;3:160025. doi:10.1038/sdata.2016.25.

22. Zook JM, Hansen NF, Olson ND, Chapman LM, Mullikin JC, Xiao C, et al. A robust benchmark for germline structural variant detection. bioRxiv. 2019:664623. doi:10.1101/664623.

23. Rausch T, Zichner T, Schlattl A, Stutz AM, Benes V and Korbel JO. DELLY: structural variant discovery by integrated paired-end and split-read analysis. Bioinformatics. 2012;28 18:i333-i9. doi:10.1093/bioinformatics/bts378.

24. Handsaker RE, Van Doren V, Berman JR, Genovese G, Kashin S, Boettger LM, et al. Large multiallelic copy number variations in humans. Nat Genet. 2015;47 3:296-303. doi:10.1038/ng.3200.

25. Layer RM: https://github.com/ryanlayer/stix (2018).

26. Antaki D, Brandler WM and Sebat J. SV2: accurate structural variation genotyping and de novo mutation detection from whole genomes. Bioinformatics. 2018;34 10:1774-7. doi:10.1093/bioinformatics/btx813.

27. Chiang C, Layer RM, Faust GG, Lindberg MR, Rose DB, Garrison EP, et al. SpeedSeq: ultra-fast personal genome analysis and interpretation. Nat Methods. 2015;12 10:966-8. doi:10.1038/nmeth.3505.

28. Li H. A statistical framework for SNP calling, mutation discovery, association mapping and population genetical parameter estimation from sequencing data. Bioinformatics. 2011;27 21:2987-93. doi:10.1093/bioinformatics/btr509.

29. Layer RM, Pedersen BS, DiSera T, Marth GT, Gertz J and Quinlan AR. GIGGLE: a search engine for large-scale integrated genome analysis. Nat Methods. 2018;15 2:123-6. doi:10.1038/nmeth.4556.

30. McKenna A, Hanna M, Banks E, Sivachenko A, Cibulskis K, Kernytsky A, et al. The Genome Analysis Toolkit: a MapReduce framework for analyzing next-generation DNA sequencing data. Genome Res. 2010;20 9:1297-303. doi:10.1101/gr.107524.110.

31. Layer RM, Chiang C, Quinlan AR and Hall IM. LUMPY: a probabilistic framework for structural variant discovery. Genome Biol. 2014;15 6:R84. doi:10.1186/gb-2014-15-6-r84.

32. Chen X, Schulz-Trieglaff O, Shaw R, Barnes B, Schlesinger F, Kallberg M, et al. Manta: rapid detection of structural variants and indels for germline and cancer sequencing applications. Bioinformatics. 2016;32 8:1220-2. doi:10.1093/bioinformatics/btv710.

33. Holtgrewe M. *Mason-A Read Simulator for Second Generation Sequencing Data*. 2010. Institut für Mathematik und Informatik, Freie Universität Berlin.

34. Li H. Aligning sequence reads, clone sequences and assembly contigs with BWA-MEM. ArXiv e-prints. 2013; doi: arXiv:1303.3997 [q-bio.GN].

35. Garrison E and Marth G. Haplotype-based variant detection from short-read sequencing. ArXiv e-prints. 2012.

36. Li H. Tabix: fast retrieval of sequence features from generic TAB-delimited files. Bioinformatics. 2011;27 5:718-9. doi:10.1093/bioinformatics/btq671.

Click here to access/download
Supplementary Material
GIGA-D-19-00035_Tables.xlsx

Response to the comments of the reviewers Click here to access/download;Personal Cover;GIGA-D-19-00035_Response.docx

Editor:

A: We thank the reviewers for their helpful suggestions and for highlighting the importance of this first side-by-side assessment of SV genotyping software. We were able to incorporate all the suggestions made and performed the suggested analysis as requested. The modified text is highlighted in red in the manuscript.

Reviewer reports:

Reviewer #1: In this manuscript Chander and colleagues compare the performance of several tools that have been developed to assess the presence of a target set of structural variants in a new sample, given an aligned sequence file and VCF as input. The introduction describes the problem in sufficient detail. The authors conclude that none of the methods is clearly superior for correctly genotyping samples. Moreover, it appears that none of the methods can be endorsed as a strong overall performer, and attempting to combine the results of several tools in a voting approach may be unwise due to either lack of coverage of certain classes of SV, or requirements that VCFs be pre-processed using specific tools. Overall, the impression is of a field grappling with a difficult problem, with tools that are not yet ready for general use by non-specialists.

The manuscript is technically well-executed. The writing requires some proof-reading.

A: We thank the reviewer for his recommendations. Yes, we agree that the field is in an early stage, which asserts the importance of such benchmarks to reveal the current state and highlight what is missing.

Major comments:

1) Figures for the accuracy of SV calling are derived from high-quality but older citations that used low-pass sequencing that was more prevalent 6-8 years ago (e.g. Mills Nature 2011). More recent studies of SV (at least in cancer) use deeper short read sequencing (e.g. 30-100x depth). These methods are applied to non-homogenous cell populations where not all cells will harbor a given SV. **The introduction would be improved if the** authors commented briefly on 1) the trade-offs between higher sequencing depth, SV calling accuracy, and cost; and 2)

the different applications of SV genotyping in a germline vs. tumor context. These factors may influence the utility of a given tool for different applications.

A: We agree with the reviewer that recent studies focus on higher sequencing depth. We did that for our simulation study (30x) and utilized even the full 300x data set for GIAB. However, we disagree that these are outdated methods. The methods we focused here are for SV genotyping and not for the initial SV discoveries. Other publications have already presented benchmarking of the latter (e.g. Kosugi et al. 2019, PMID: 31159850; Sedlazeck et. al. 2018 and Nattestad et. al 2018, PMID: 29713083 and 29954844).

2) Table 1 should distinguish between tools that are agnostic to the SV calling tool and those such as Delly or SVTyper that require a VCF generated using a specific SV method.

A: We thank the reviewer for this suggestion and have modified the table accordingly.

3) It's not clear what lines 125-126 mean, "given the nature of the data". Please be explicit about results were expected, why they were expected, and the degree to which the observed results conformed to those expectations.

A: We have clarified that by this statement- "We discovered only 17 false positive calls after the initial SV discovery. This low number of false positives is in contrast to reports from other studies. However, we are using here simulated data which does not take into account the complexities involved in regions of SVs and other sequencing biases. Interestingly, while this simulated data set represents an ideal case we still missed around 17.25% of the simulated SVs.
" at line 133

4) The authors test whether the SV genotypers fail to call incorrectly genotyped SVs; this seems a distinct task from whether they correctly report the absence of a given SV when it is truly not present in the sample.

A: In this benchmark, we have assessed true positives (SVs that are present and should be re-found by the SV genotypers), false positives (SVs that are present in the input VCF but not present in the sample) and false negatives (SVs that are

present in the sample and in the input VCF but were not re – identified). The case where SVs are provided in the input VCF but are not supported in the sample is reflected as false positives test cases. The case where reads don't show any significant distortion in this region is trivial and thus was not explicitly assessed.

Reviewer #2: This study is designed to evaluate tools for the genotyping or validation of structural variant calls, with regard to their accuracy, applicability to types of structural variant, and usability. The authors make a strong case for the importance of this evaluation, due to the high false positive rates of most structural variant calling techniques that rely on short read sequencing technology and the utility of genotyping SVs, as well as counting alleles for population-level studies. SV genotypers were evaluated against a set of simulated SVs of different types, then against a set of SVs identified in a real sample through the use of many different and complementary technologies and methods by the GIAB consortium. The conclusion of this study is that while SV genotypers can be used to improve the accuracy of SV calls, they require considerable enhancement in usability and general applicability.

I like the simulation experiment, but the analysis needs to be improved. First, the figure. The axes are not labeled, and the colors are not described. The yellow and the orange are so similar, and the plots are so small that I didn't realize they were different colors until I zoomed way in. I kept getting lost in the description about which SVs are supported by which method.

A: We thank the reviewer for this suggestion. We have followed the guidelines for preparing the figures and used colors that are suggested for color blind people. The figure is meant to give a general trend showing in green/orange and red for the different performance abilities of the SV genotypers. The precise numbers are provided in Supplementary Tables.

It may be worth using some other visual cue (maybe another color) to indicated that a particular method does not work for a particular SV type, instead of just saying it genotypes 0% of the SVs. For example, SVTYPER supports BNDs but just misses all of them while GenomeSTRIP doesn't even try to genotype BNDs. That different is important and it would be helpful if it was clearer.

A: We discussed these limitations in the introduction of the manuscript and now this is also highlighted in Table 1. Figure 1 represents the ability to call certain SV types and sizes, from a standard VCF file. To clarify the presentation, we removed the BND assessment from Figure1 since none of the methods were able to successfully provide those genotypes. In the example of SVTyper, it cannot call BND events since it requires specific input data provided only by Lumpy. For other SV types we have included tags that we could reproduce (e.g. CIPOS, CIEND) in the VCF files to enable a comparison. In contrast, GenomeSTRIP indeed only focuses on DEL/ DUP events, which is similar to SV2.

In the description, since the overall rates are so dependent on the supported SV types, it may be worth reorganizing this section around SV types instead of going through each method and given a single rate (e.g., for DEL the method A was x%, B was y% and C doesn't do DEL).

A: We have made minor modifications to the text, since we were motivated to illustrate the overall combined performance of the methods. Figure 1 already illustrates the different advantages and disadvantages of the individual methods based on the different types and size regimes.

Question on the simulation experiment. Were the events all HET?

A: The SVs were all homozygous. We have clarified this in the manuscript.

Why only test the events that were detected? I get that in a non-simulated scenario you will only test the SVs that you detect, but it would be interesting to test how/if undetected SVs can be genotyped. This is a claim that has been made from long-read sequencing and it seems you can test it here too.

A: Thank you for raising this point. We had these tests included over the GIAB data set where a multitude of SV were only detectable using long reads. We highlighted the ability of SVgenotypers to identify these events. Furthermore, in the simulation we benchmarked the case where there were false SV calls and the ability of the SV genotypers to detect these.

On line 147, I don't think you meant "filter out falsely called SVs." That part is about true positives. The next paragraph is about filtering false positives.

A: We have modified the main text to clarify this point. This was one of the points we assessed in the benchmarks. We used standard SV callers (Delly, Lumpy and Manta) over a union set to obtain SV calls over each simulated data set. This also included a 17 falsely called SVs due to mapping errors or other reasons. We used these 17 artifacts to benchmark how these SV genotypers perform over a false indication of an SV in the sample. This could, for example, represent regions that are repetitive or otherwise challenging.

In the false positive part, you say that STIX does better than SVTYPER, but the numbers given do not seem to support that. STIX filters 76.47% and SVTYPER filters 81.82%. I am guessing the 81.82 is typo since you can't get to that number with 17 as a denominator.

A: We apologize for this confusion. The numbers reported in the Supplementary table were correct. We corrected this sentence: "… Genome STRiP performed best with filtering out all falsely detected SVs, but suffers from the lowest ability to genotype SV variation. STIX performed better as it can filter out 13 (76.4 %) of the false positive SV calls. In contrast, STIX also achieved a higher (71.76%) performance for correctly identifying SVs. Although SVTyper had the highest accurately genotyped SVs, it filtered out less of the false positives (69.70%) obtained during the discovery phase."

The dependence that some methods have on particular VCF flags is interesting, but I think you should comment on if either meet the VCF spec.

A: We clarified this in the main text. The issue is that all the input VCFs conform to the expected standard, but many tools require additional flags, which are not provided by other methods and are not easily reproducible.

This study, like most which deal with SV detection methods, suffered from a lack of fully reliable positive controls. The combination of simulated data and highly vetted GIAB SV calls provide a likely best currently possible answer to that problem. The low number of false positive SV calls in the simulated data suggest that the simulation was a best-case scenario for SV calling and therefore genotyping. Testing against a curated set of known false calls from previous published work might provide a useful complementary test of how well the

genotypers handle false positives.

A: We appreciate the comment – and the recognition of the difficulty of providing a ‘gold standard’ for this kind of work. In the simulation, we focused on the SVs that are falsely called but are in regions that show mapping errors. The other cases, as suggested here, would be exemplified by an SV in an input VCF vs. non-altered mapping within specified regions. These cases are easily distinguishable by e.g. a lack of abnormally mapped reads and thus we did not assess this.

Use of the GIAB SV callset as a second test case for the genotypers is a valuable exercise and demonstrates the performance of these genotypers in real data. A mostly unavoidable source of concern is the reliability of the calls from GIAB that are used in this experiment. These calls are an attempt to sensitively identify all structural variation in the Ashkenazi Son sample and seem likely (due to the number of events) to contain a large number of false positives. This could be reflected in the number of variants that were not detected by any of the genotypers, but those could also represent real variants that genotypers could not identify. It would therefore strengthen the argument to have some additional analysis of the variants that were not identified by any genotyper, such as a downsampling and visual review. If the majority of those variants appear to be false positives in GIAB rather than false negatives in genotyping, the performance of the genotypers may potentially be much stronger than it currently appears to be.

A: We agree with the reviewer that this could have been a potential pitfall. However, the GIAB calls in v0.5 have been produced via multiple rounds of manual curation using various sequencing technologies and assembly and mapping approaches. The combined high confidence set within the high confidence regions indeed represents a highly accurate SV call set that has been assessed multiple times by us and others over various studies. Hence, we do not share this concern.

How dependent is the performance of STIX on finding just one read supporting an SV?

A: As we highlighted in the main text, it is a disadvantage of STIX to only report read counts vs. genotypes from other methods. Since this is a limitation of the method itself, we can just highlight this in the discussion as we did.

A missing piece for all of the experiments is runtime. Is one of these more efficient than the others?

A: Thank you for this suggestion. We have included the average CPU time measured over 20 runs as Supplementary Table. STIX is the fastest method (0.4 seconds) followed by Delly (3.7s) and SVtyper (9.6s). The slowest by far is GenomeSTRiP (33.8 min).
