# Supplementary material for: Evaluation of computational genotyping of structural variation for clinical diagnoses
Source: Gigascience. 2019 Sep 8;8(9):giz110. doi: 10.1093/gigascience/giz110 (PMC6732172; doi:10.1093/gigascience/giz110)
Supplement: giz110_Response_to_Reviewer_Comments_Original_Submission [file giz110_response_to_reviewer_comments_original_submission.pdf]

Author's Response To Reviewer Comments

Close

A: We thank the reviewers for their helpful suggestions and for highlighting the importance of this first side-by-side assessment of SV genotyping software. We were able to incorporate all the suggestions made and performed the suggested analysis as requested. The modified text is highlighted in red in the manuscript.

Reviewer reports:

Reviewer #1: In this manuscript Chander and colleagues compare the performance of several tools that have been developed to assess the presence of a target set of structural variants in a new sample, given an aligned sequence file and VCF as input. The introduction describes the problem in sufficient detail. The authors conclude that none of the methods is clearly superior for correctly genotyping samples. Moreover, it appears that none of the methods can be endorsed as a strong overall performer, and attempting to combine the results of several tools in a voting approach may be unwise due to either lack of coverage of certain classes of SV, or requirements that VCFs be pre-processed using specific tools. Overall, the impression is of a field grappling with a difficult problem, with tools that are not yet ready for general use by non-specialists.

The manuscript is technically well-executed. The writing requires some proof-reading.

A: We thank the reviewer for his recommendations. Yes, we agree that the field is in an early stage, which asserts the importance of such benchmarks to reveal the current state and highlight what is missing.

Major comments:

1) Figures for the accuracy of SV calling are derived from high-quality but older citations that used low-pass sequencing that was more prevalent 6-8 years ago (e.g. Mills Nature 2011). More recent studies of SV (at least in cancer) use deeper short read sequencing (e.g. 30-100x depth). These methods are applied to non-homogenous cell populations where not all cells will harbor a given SV. The introduction would be improved if the authors commented briefly on 1) the trade-offs between higher sequencing depth, SV calling accuracy, and cost; and 2) the different applications of SV genotyping in a germline vs. tumor context. These factors may influence the utility of a given tool for different applications.

A: We agree with the reviewer that recent studies focus on higher sequencing depth. We did that for our simulation study (30x) and utilized even the full 300x data set for GIAB. However, we disagree that these are outdated methods. The methods we focused here are for SV genotyping and not for the initial SV discoveries. Other publications have already presented benchmarking of the latter (e.g. Kosugi et al. 2019, PMID: 31159850; Sedlazeck et. al. 2018 and Nattestad et. al 2018, PMID: 29713083 and 29954844).

2) Table 1 should distinguish between tools that are agnostic to the SV calling tool and those such as Delly or SVTyper that require a VCF generated using a specific SV method.

A: We thank the reviewer for this suggestion and have modified the table accordingly.

3) It's not clear what lines 125-126 mean, "given the nature of the data". Please be explicit about results were expected, why they were expected, and the degree to which the observed results conformed to those expectations.

A: We have clarified that by this statement- “We discovered only 17 false positive calls after the initial SV discovery. This low number of false positives is in contrast to reports from other studies. However, we are using here simulated data which does not take into account the complexities involved in regions of SVs and other sequencing biases. Interestingly, while this simulated data set represents an ideal case

we still missed around 17.25% of the simulated SVs.
" at line 133

4) The authors test whether the SV genotypers fail to call incorrectly genotyped SVs; this seems a distinct task from whether they correctly report the absence of a given SV when it is truly not present in the sample.

A: In this benchmark, we have assessed true positives (SVs that are present and should be re-found by the SV genotypers), false positives (SVs that are present in the input VCF but not present in the sample) and false negatives (SVs that are present in the sample and in the input VCF but were not re – identified). The case where SVs are provided in the input VCF but are not supported in the sample is reflected as false positives test cases. The case where reads don't show any significant distortion in this region is trivial and thus was not explicitly assessed.

Reviewer #2: This study is designed to evaluate tools for the genotyping or validation of structural variant calls, with regard to their accuracy, applicability to types of structural variant, and usability. The authors make a strong case for the importance of this evaluation, due to the high false positive rates of most structural variant calling techniques that rely on short read sequencing technology and the utility of genotyping SVs, as well as counting alleles for population-level studies. SV genotypers were evaluated against a set of simulated SVs of different types, then against a set of SVs identified in a real sample through the use of many different and complementary technologies and methods by the GIAB consortium. The conclusion of this study is that while SV genotypers can be used to improve the accuracy of SV calls, they require considerable enhancement in usability and general applicability.

I like the simulation experiment, but the analysis needs to be improved. First, the figure. The axes are not labeled, and the colors are not described. The yellow and the orange are so similar, and the plots are so small that I didn't realize they were different colors until I zoomed way in. I kept getting lost in the description about which SVs are supported by which method.

A: We thank the reviewer for this suggestion. We have followed the guidelines for preparing the figures and used colors that are suggested for color blind people. The figure is meant to give a general trend showing in green/orange and red for the different performance abilities of the SV genotypers. The precise numbers are provided in Supplementary Tables.

It may be worth using some other visual cue (maybe another color) to indicated that a particular method does not work for a particular SV type, instead of just saying it genotypes 0% of the SVs. For example, SVTYPER supports BNDs but just misses all of them while GenomeSTRIP doesn't even try to genotype BNDs. That different is important and it would be helpful if it was clearer.

A: We discussed these limitations in the introduction of the manuscript and now this is also highlighted in Table 1. Figure 1 represents the ability to call certain SV types and sizes, from a standard VCF file. To clarify the presentation, we removed the BND assessment from Figure1 since none of the methods were able to successfully provide those genotypes. In the example of SVTyper, it cannot call BND events since it requires specific input data provided only by Lumpy. For other SV types we have included tags that we could reproduce (e.g. CIPOS, CIEND) in the VCF files to enable a comparison. In contrast, GenomeSTRIP indeed only focuses on DEL/ DUP events, which is similar to SV2.

In the description, since the overall rates are so dependent on the supported SV types, it may be worth reorganizing this section around SV types instead of going through each method and given a single rate (e.g., for DEL the method A was x%, B was y% and C doesn't do DEL).

A: We have made minor modifications to the text, since we were motivated to illustrate the overall combined performance of the methods. Figure 1 already illustrates the different advantages and disadvantages of the individual methods based on the different types and size regimes.

Question on the simulation experiment. Were the events all HET?

A: The SVs were all homozygous. We have clarified this in the manuscript.

Why only test the events that were detected? I get that in a non-simulated scenario you will only test the SVs that you detect, but it would be interesting to test how/if undetected SVs can be genotyped.

This is a claim that has been made from long-read sequencing and it seems you can test it here too.

A: Thank you for raising this point. We had these tests included over the GIAB data set where a multitude of SV were only detectable using long reads. We highlighted the ability of SVgenotypers to identify these events. Furthermore, in the simulation we benchmarked the case where there were false SV calls and the ability of the SV genotypers to detect these.

On line 147, I don't think you meant "filter out falsely called SVs." That part is about true positives. The next paragraph is about filtering false positives.

A: We have modified the main text to clarify this point. This was one of the points we assessed in the benchmarks. We used standard SV callers (Delly, Lumpy and Manta) over a union set to obtain SV calls over each simulated data set. This also included a 17 falsely called SVs due to mapping errors or other reasons. We used these 17 artifacts to benchmark how these SV genotypers perform over a false indication of an SV in the sample. This could, for example, represent regions that are repetitive or otherwise challenging.

In the false positive part, you say that STIX does better than SVTYPER, but the numbers given do not seem to support that. STIX filters 76.47% and SVTYPER filters 81.82%. I am guessing the 81.82 is typo since you can't get to that number with 17 as a denominator.

A: We apologize for this confusion. The numbers reported in the Supplementary table were correct. We corrected this sentence: "… Genome STRiP performed best with filtering out all falsely detected SVs, but suffers from the lowest ability to genotype SV variation. STIX performed better as it can filter out 13 (76.4 %) of the false positive SV calls. In contrast, STIX also achieved a higher (71.76%) performance for correctly identifying SVs. Although SVTyper had the highest accurately genotyped SVs, it filtered out less of the false positives (69.70%) obtained during the discovery phase."

The dependence that some methods have on particular VCF flags is interesting, but I think you should comment on if either meet the VCF spec.

A: We clarified this in the main text. The issue is that all the input VCFs conform to the expected standard, but many tools require additional flags, which are not provided by other methods and are not easily reproducible.

This study, like most which deal with SV detection methods, suffered from a lack of fully reliable positive controls. The combination of simulated data and highly vetted GIAB SV calls provide a likely best currently possible answer to that problem. The low number of false positive SV calls in the simulated data suggest that the simulation was a best-case scenario for SV calling and therefore genotyping. Testing against a curated set of known false calls from previous published work might provide a useful complementary test of how well the genotypers handle false positives.

A: We appreciate the comment – and the recognition of the difficulty of providing a 'gold standard' for this kind of work. In the simulation, we focused on the SVs that are falsely called but are in regions that show mapping errors. The other cases, as suggested here, would be exemplified by an SV in an input VCF vs. non-altered mapping within specified regions. These cases are easily distinguishable by e.g. a lack of abnormally mapped reads and thus we did not assess this.

Use of the GIAB SV callset as a second test case for the genotypers is a valuable exercise and demonstrates the performance of these genotypers in real data. A mostly unavoidable source of concern is the reliability of the calls from GIAB that are used in this experiment. These calls are an attempt to sensitively identify all structural variation in the Ashkenazi Son sample and seem likely (due to the number of events) to contain a large number of false positives. This could be reflected in the number of variants that were not detected by any of the genotypers, but those could also represent real variants that genotypers could not identify. It would therefore strengthen the argument to have some additional analysis of the variants that were not identified by any genotyper, such as a downsampling and visual review. If the majority of those variants appear to be false positives in GIAB rather than false negatives in genotyping, the performance of the genotypers may potentially be much stronger than it currently appears to be.

A: We agree with the reviewer that this could have been a potential pitfall. However, the GIAB calls in v0.5 have been produced via multiple rounds of manual curation using various sequencing technologies

and assembly and mapping approaches. The combined high confidence set within the high confidence regions indeed represents a highly accurate SV call set that has been assessed multiple times by us and others over various studies. Hence, we do not share this concern.

How dependent is the performance of STIX on finding just one read supporting an SV?

A: As we highlighted in the main text, it is a disadvantage of STIX to only report read counts vs. genotypes from other methods. Since this is a limitation of the method itself, we can just highlight this in the discussion as we did.

A missing piece for all of the experiments is runtime. Is one of these more efficient than the others?

A: Thank you for this suggestion. We have included the average CPU time measured over 20 runs as Supplementary Table. STIX is the fastest method (0.4 seconds) followed by Delly (3.7s) and SVtyper (9.6s). The slowest by far is GenomeSTRiP (33.8 min).

Close
