# Supplementary material for: Evaluation of computational genotyping of structural variation for clinical diagnoses
Source: Gigascience. 2019 Sep 8;8(9):giz110. doi: 10.1093/gigascience/giz110 (PMC6732172; doi:10.1093/gigascience/giz110)
Supplement: giz110_Response_to_Reviewer_Comments_Revision_1 [file giz110_response_to_reviewer_comments_revision_1.pdf]

Author's Response To Reviewer Comments

Close

We have uploaded the required information to GigaDB http://dx.doi.org/10.5524/100641 and made the requested changes in the data availability section.

Close
