# Supplementary material for: Evaluation of computational genotyping of structural variation for clinical diagnoses
Source: Gigascience. 2019 Sep 8;8(9):giz110. doi: 10.1093/gigascience/giz110 (PMC6732172; doi:10.1093/gigascience/giz110)
Supplement: giz110_Reviewer_1_Report_Original_Submission — David Quigley -- 2/28/2019 Reviewed [file giz110_reviewer_1_report_original_submission.pdf]

Reviewer Report

Title: Evaluation of computational genotyping of Structural Variations for clinical diagnoses

Version: Original Submission **Date:** 2/28/2019

Reviewer name: David Quigley

Reviewer Comments to Author:

In this manuscript Chander and colleagues compare the performance of several tools that have been developed to assess the presence of a target set of structural variants in a new sample, given an aligned sequence file and VCF as input. The introduction describes the problem in sufficient detail. The authors conclude that none of the methods is clearly superior for correctly genotyping samples. Moreover, it appears that none of the methods can be endorsed as a strong overall performer, and attempting to combine the results of several tools in a voting approach may be unwise due to either lack of coverage of certain classes of SV, or requirements that VCFs be pre-processed using specific tools. Overall, the impression is of a field grappling with a difficult problem, with tools that are not yet ready for general use by non-specialists.

The manuscript is technically well-executed. The writing requires some proof-reading.

Major comments:

1) Figures for the accuracy of SV calling are derived from high-quality but older citations that used low-pass sequencing that was more prevalent 6-8 years ago (e.g. Mills Nature 2011). More recent studies of SV (at least in cancer) use deeper short read sequencing (e.g. 30-100x depth). These methods are applied to non-homogenous cell populations where not all cells will harbor a given SV. The introduction would be improved if the authors commented briefly on 1) the trade-offs between higher sequencing depth, SV calling accuracy, and cost; and 2) the different applications of SV genotyping in a germline vs. tumor context. These factors may influence the utility of a given tool for different applications.

2) Table 1 should distinguish between tools that are agnostic to the SV calling tool and those such as Delly or SVTyper that require a VCF generated using a specific SV method.

3) It's not clear what lines 125-126 mean, "given the nature of the data". Please be explicit about results were expected, why they were expected, and the degree to which the observed results conformed to those expectations.

4) What do the colors indicate in figure 1?

5) The authors test whether the SV genotypers fail to call incorrectly genotyped SVs; this seems a distinct task from whether they correctly report the absence of a given SV when it is truly not present in the sample.

Minor comments

1) It is fair to say that SV methods are still maturing, but it is perhaps rather strong to say that they are in their infancy; Pindel was published in 2009 and DELLY was published in 2012. Perhaps it would be more accurate to say that SV genotyping methods are just emerging.

2) The manuscript should be edited. Line 97 says "translations", I think you mean "translocations". Line

133 and 286, you mean "custom" not "costume"; line 204 you don't likely mean "size regimes".

Methods

Are the methods appropriate to the aims of the study, are they well described, and are necessary controls included? Choose an item.

Conclusions

Are the conclusions adequately supported by the data shown? Choose an item.

Reporting Standards

Does the manuscript adhere to the journal's guidelines on minimum standards of reporting? Choose an item.

Choose an item.

Statistics

Are you able to assess all statistics in the manuscript, including the appropriateness of statistical tests used? Choose an item.

Quality of Written English

Please indicate the quality of language in the manuscript: Choose an item.

Declaration of Competing Interests

Please complete a declaration of competing interests, considering the following questions:

- Have you in the past five years received reimbursements, fees, funding, or salary from an organisation that may in any way gain or lose financially from the publication of this manuscript, either now or in the future?
- Do you hold any stocks or shares in an organisation that may in any way gain or lose financially from the publication of this manuscript, either now or in the future?
- Do you hold or are you currently applying for any patents relating to the content of the manuscript?
- Have you received reimbursements, fees, funding, or salary from an organization that holds or has applied for patents relating to the content of the manuscript?
- Do you have any other financial competing interests?
- Do you have any non-financial competing interests in relation to this paper?

If you can answer no to all of the above, write 'I declare that I have no competing interests' below. If your reply is yes to any, please give details below.

I declare that I have no competing interests.

I agree to the open peer review policy of the journal. I understand that my name will be included on my report to the authors and, if the manuscript is accepted for publication, my named report including any attachments I upload will be posted on the website along with the authors' responses. I agree for my report to be made available under an Open Access Creative Commons CC-BY license (http://creativecommons.org/licenses/by/4.0/). I understand that any comments which I do not wish to be included in my named report can be included as confidential comments to the editors, which will not be published.

Choose an item.

To further support our reviewers, we have joined with Publons, where you can gain additional credit to further highlight your hard work (see: https://publons.com/journal/530/gigascience). On publication of this paper, your review will be automatically added to Publons, you can then choose whether or not to claim your Publons credit. I understand this statement.

Yes Choose an item.
