# Supplementary material for: Evaluation of computational genotyping of structural variation for clinical diagnoses
Source: Gigascience. 2019 Sep 8;8(9):giz110. doi: 10.1093/gigascience/giz110 (PMC6732172; doi:10.1093/gigascience/giz110)
Supplement: giz110_Reviewer_2_Report_Original_Submission — Ryan Layer -- 3/19/2019 Reviewed [file giz110_reviewer_2_report_original_submission.pdf]

Reviewer Report

Title: Evaluation of computational genotyping of Structural Variations for clinical diagnoses

Version: Original Submission **Date:** 3/19/2019

Reviewer name: Ryan Layer

Reviewer Comments to Author:

This study is designed to evaluate tools for the genotyping or validation of structural variant calls, with regard to their accuracy, applicability to types of structural variant, and usability. The authors make a strong case for the importance of this evaluation, due to the high false positive rates of most structural variant calling techniques that rely on short read sequencing technology and the utility of genotyping SVs, as well as counting alleles for population-level studies. SV genotypers were evaluated against a set of simulated SVs of different types, then against a set of SVs identified in a real sample through the use of many different and complementary technologies and methods by the GIAB consortium. The conclusion of this study is that while SV genotypers can be used to improve the accuracy of SV calls, they require considerable enhancement in usability and general applicability.

I like the simulation experiment, but the analysis needs to be improved. First, the figure. The axes are not labeled, and the colors are not described. The yellow and the orange are so similar, and the plots are so small that I didn't realize they were different colors until I zoomed way in. I kept getting lost in the description about which SVs are supported by which method. It may be worth using some other visual cue (maybe another color) to indicated that a particular method does not work for a particular SV type, instead of just saying it genotypes 0% of the SVs. For example, SVTYPER supports BNDs but just misses all of them while GenomeSTRIP doesn't even try to genotype BNDs. That different is important and it would be helpful if it was clearer. In the description, since the overall rates are so dependent on the supported SV types, it may be worth reorganizing this section around SV types instead of going through each method and given a single rate (e.g., for DEL the method A was x%, B was y% and C doesn't do DEL).

Question on the simulation experiment. Were the events all HET? Why only test the events that were detected? I get that in a non-simulated scenario you will only test the SVs that you detect, but it would be interesting to test how/if undetected SVs can be genotyped. This is a claim that has been made from long-read sequencing and it seems you can test it here too.

On line 147, I don't think you meant "filter out falsely called SVs." That part is about true positives. The next paragraph is about filtering false positives.

In the false positive part, you say that STIX does better than SVTYPER, but the numbers given do not seem to support that. STIX filters 76.47% and SVTYPER filters 81.82%. I am guessing the 81.82 is typo since you can't get to that number with 17 as a denominator.

The dependence that some methods have on particular VCF flags is interesting, but I think you should comment on if either meet the VCF spec.

This study, like most which deal with SV detection methods, suffered from a lack of fully reliable positive controls. The combination of simulated data and highly vetted GIAB SV calls provide a likely best

currently possible answer to that problem. The low number of false positive SV calls in the simulated data suggest that the simulation was a best-case scenario for SV calling and therefore genotyping. Testing against a curated set of known false calls from previous published work might provide a useful complementary test of how well the genotypers handle false positives.
Use of the GIAB SV callset as a second test case for the genotypers is a valuable exercise and demonstrates the performance of these genotypers in real data. A mostly unavoidable source of concern is the reliability of the calls from GIAB that are used in this experiment. These calls are an attempt to sensitively identify all structural variation in the Ashkenazi Son sample and seem likely (due to the number of events) to contain a large number of false positives. This could be reflected in the number of variants that were not detected by any of the genotypers, but those could also represent real variants that genotypers could not identify. It would therefore strengthen the argument to have some additional analysis of the variants that were not identified by any genotyper, such as a downsampling and visual review. If the majority of those variants appear to be false positives in GIAB rather than false negatives in genotyping, the performance of the genotypers may potentially be much stronger than it currently appears to be.
How dependent is the performance of STIX on finding just one read supporting an SV?
A missing piece for all of the experiments is runtime. Is one of these more efficient than the others?

Methods

Are the methods appropriate to the aims of the study, are they well described, and are necessary controls included? Choose an item.

Conclusions

Are the conclusions adequately supported by the data shown? Choose an item.

Reporting Standards

Does the manuscript adhere to the journal's guidelines on minimum standards of reporting? Choose an item.

Choose an item.

Statistics

Are you able to assess all statistics in the manuscript, including the appropriateness of statistical tests used? Choose an item.

Quality of Written English

Please indicate the quality of language in the manuscript: Choose an item.

Declaration of Competing Interests

Please complete a declaration of competing interests, considering the following questions:

- Have you in the past five years received reimbursements, fees, funding, or salary from an organisation that may in any way gain or lose financially from the publication of this manuscript, either now or in the future?
- Do you hold any stocks or shares in an organisation that may in any way gain or lose financially from the publication of this manuscript, either now or in the future?
- Do you hold or are you currently applying for any patents relating to the content of the manuscript?
- Have you received reimbursements, fees, funding, or salary from an organization that holds or has applied for patents relating to the content of the manuscript?
- Do you have any other financial competing interests?
- Do you have any non-financial competing interests in relation to this paper?

If you can answer no to all of the above, write 'I declare that I have no competing interests' below. If your reply is yes to any, please give details below.

I declare that I have no competing interests

I agree to the open peer review policy of the journal. I understand that my name will be included on my report to the authors and, if the manuscript is accepted for publication, my named report including any attachments I upload will be posted on the website along with the authors' responses. I agree for my report to be made available under an Open Access Creative Commons CC-BY license (http://creativecommons.org/licenses/by/4.0/). I understand that any comments which I do not wish to be included in my named report can be included as confidential comments to the editors, which will not be published.

Choose an item.

To further support our reviewers, we have joined with Publons, where you can gain additional credit to further highlight your hard work (see: https://publons.com/journal/530/gigascience). On publication of this paper, your review will be automatically added to Publons, you can then choose whether or not to claim your Publons credit. I understand this statement.

Yes Choose an item.
